# Site‐Specific Antibody Assembly on Nanoparticles via a Versatile Coating Method for Improved Cell Targeting

**DOI:** 10.1002/advs.202206546

**Published:** 2023-01-25

**Authors:** Qianyi Zhang, Jieying Liang, Andre Bongers, Joseph J. Richardson, Kang Liang, Zi Gu

**Affiliations:** ^1^ School of Chemical Engineering University of New South Wales Sydney NSW 2052 Australia; ^2^ Australian Centre for NanoMedicine (ACN) University of New South Wales Sydney NSW 2052 Australia; ^3^ Biological Resources Imaging Laboratory Mark wainwright Analytical Centre The University of New South Wales Sydney NSW 2052 Australia; ^4^ School of Engineering RMIT University Melbourne Victoria 3000 Australia; ^5^ Graduate School of Biomedical Engineering The University of New South Wales Sydney NSW 2052 Australia; ^6^ UNSW RNA Institute University of New South Wales Sydney NSW 2052 Australia

**Keywords:** antibody conjugation, metal–organic frameworks (MOF), targeted delivery, tumor therapy, bioimaging

## Abstract

Antibody‐nanoparticle conjugates are promising candidates for precision medicine. However, developing a controllable method for conjugating antibodies to nanoparticles without compromising the antibody activity represents a critical challenge. Here, a facile and generalizable film‐coating method is presented using zeolitic imidazole framework‐8 (ZIF‐8) to immobilize antibodies on various nanoparticles in a favorable orientation for enhanced cell targeting. Different model and therapeutic antibodies (e.g., Herceptin) are assembled on nanoparticles via a biomineralized film‐coating method and exhibited high antibody loading and targeting efficiencies. Importantly, the antibodies selectively bind to ZIF‐8 via their Fc regions, which favorably exposes the functional Fab regions to the biological target, thus improving the cell targeting ability of antibody‐coated nanoparticles. In combination, molecular dynamics simulations and experimental studies on antibody immobilization, orientation efficiency, and biofunctionality collectively demonstrate that this versatile site‐specific antibody conjugation method provides effective control over antibody orientation and leads to improved cell targeting for a variety of nanoparticles.

## Graphical abstract

A film‐coating method by using ZIF‐8 to immobilize antibodies on various nanoparticles in a favorable orientation is developed for cell targeting. The underlying mechanism of orientated antibody conjugation is revealed to be unique site‐selective binding of antibodies to ZIF‐8. Its broad applicability is demonstrated by conjugating antibodies on a range of nanoparticles to realize cell‐specific targeting for potential biomedical applications.

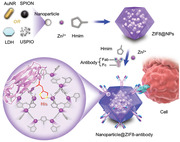

## 1 Introduction

Functionalizing nanoparticles using cell‐specific ligands have been explored to concentrate nanoparticles at a biological target, thereby enabling high therapeutic efficacy and low off‐target toxicity.^[^ ^1^ ^]^ Cell‐specific ligands that have been conjugated to nanoparticles include small molecules, sugars, peptides, proteins, nucleic acids, and antibodies.^[^ ^2^ ^]^ Among these ligands, antibodies confer unique advantages such as being highly specific, approved as therapeutics themselves, and offering selective recognition capacity.^[^ ^3^ ^]^ Immunoglobulin G (IgG) is a specific class of antibodies regularly used as a therapeutic and targeting agent as it can bind to specific cell receptors through highly specific fragment antigen binding (Fab) regions.^[^ ^4^ ^]^ In other words, the Fab region contains the binding sites and needs to be unmodified and unhindered in order to recognize and interact with the receptors on target cells. Therefore, therapeutic or diagnostic nanoparticles should be conjugated to antibodies via the highly conserved fragment crystallizable (Fc) region, thus having limited impact on targeting.^[^ ^5^ ^]^ However, it remains a critical challenge to develop a facile and generalizable method that enables site‐selective conjugation of antibodies to nanoparticles without compromising the activity of the Fab regions.

Physical adsorption is one of simplest conjugation methods to anchor antibodies to nanoparticles via either Van der Waals forces, hydrogen bonding, hydrophobic, or electrostatic interactions. However, direct physical adsorption between antibodies and nanoparticles suffers from poor reproducibility, possible detachment at the off‐target site, and reduced targeting efficiency due to the random orientation during adsorption.^[^ ^6^ ^]^ Covalent conjugation, for example, via ethyl(dimethylaminopropyl)carbodiimide–*N*‐hydroxysuccinimide (EDC–NHS) coupling,^[^ ^7^ ^]^ maleimide–thiol coupling,^[^ ^8^ ^]^ or click reactions,^[^ ^9^ ^]^ offers relatively robust attachment. Although covalent conjugation is highly stable, the chemical modification of antibodies and nanoparticles typically requires the use of harsh chemicals, which can result in the loss of antibody functionality and can damage the conjugated material.^[^ ^10^ ^]^ Most importantly, neither physical adsorption nor chemical conjugation can control the orientation of antibodies, as they can modify or block the active sites (Fab regions), which leads to reduced cell binding efficiency. An alternative conjugation strategy is to use Fc‐binding proteins (i.e., Protein A, Protein G, Protein A/G, and Fc receptors) as a linker between the antibodies and nanoparticles, thus preserving the activity of the unbound Fab regions.^[^ ^11^ ^]^ However, these Fc‐binding proteins are expensive, and as the conjugation is biological in nature, the bound antibodies can be reversely dissociated from Fc‐binding proteins, thus limiting their applications.^[^ ^12^ ^]^ Alternatively, a recent study presented the formation of metal–organic framework (MOF) microparticles containing antibodies with controlled orientation via the accumulation of metal ions at the negatively charged Fc region.^[^ ^13^ ^]^ To date, a robust and versatile coating method to conjugate oriented antibodies on nanoparticle surface does not exist, despite the immense promise for biomedical applications.

**Figure 1:**
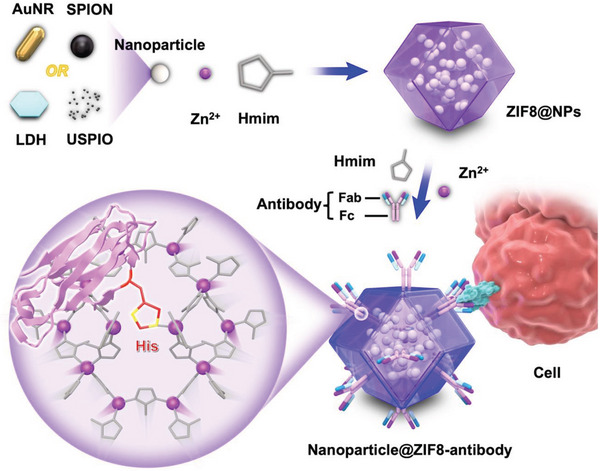
Schematic illustration of the site‐specific antibody coating on functional nanoparticles (gold nanorods (AuNR), layered double hydroxide (LDH), superparamagnetic iron oxide nanoparticles (SPION), and ultrasmall superparamagnetic iron oxide (USPIO)) via the interaction between the histidine‐rich Fc region of antibodies and Zn‐based ZIF‐8 for improved cell targeting.

Some transition metal ions (e.g., Zn^2+^, Cu^2+^, Co^2+^) have been found to preferentially bind to histidine‐rich domains in the Fc region of antibodies via coordination bonding between the metal ions and the imidazole side group of histidine.^[^ ^14^ ^]^ Similar coordination bonding is presented in zeolitic imidazole framework‐8 (ZIF‐8), a type of MOF where 2‐methylimidazol (Hmim) ligands bind to Zn^2+^ and self‐assemble into a zeolitic topological network.^[^ ^15^ ^]^ Such coordination complexes are not restricted to forming particles but can also result in thin films through careful tuning of the concentrations and ratios of ligands and metal ions.^[^ ^16^ ^]^ The similarity in these two coordination bonding systems led us to explore a new, generalized method of using ZIF‐8 to immobilize antibodies on nanoparticles with a favorable orientation. Herein, for the first time, we coated oriented antibody‐ZIF‐8 (ZIF8‐Ab) films on nanoparticles for enhanced cell targeting and biomedical applications. Firstly, MOF‐Ab films were coated onto nanoparticles via a one‐pot method, where we found that the metal type did not greatly influence the antibody conjugation efficiency, but did influence antibody orientation. Specifically, conjugation using Zn‐based MOFs showed at least 3‐fold increase in available Fab regions compared to Co, Tb, or Cu‐based MOFs. Next, we used molecular dynamics (MD) simulations to explore the binding affinity between ZIF‐8 and IgG, and found that ZIF‐8 had strong binding affinity to histidine‐rich Fc region of IgG. To demonstrate the wide potential of this coating strategy, we applied the ZIF8‐Ab coatings to a range of functional nanoparticles with different shapes, sizes, chemical compositions, and functionalities, and studied the antibody immobilization and orientation efficiency compared to traditional EDC‐NHS conjugation. Finally, the cell targeting ability of Herceptin‐based ZIF8‐Ab was remarkably higher than bare particles and particles functionalized with antibodies using EDC‐NHS conjugation in BT‐474 cells (HER2 positive). Through these studies, we established a rapid, robust, and generic thin‐film coating technology with site‐specific antibody conjugation on a range of functional nanoparticles to realize efficient targeting for potential applications in disease treatment and imaging diagnosis (**Figure** **1**). This unique one‐step coating technology for orientated antibody conjugation on nanoparticles has three key features: 1) it is a facile strategy to orient antibodies without the addition of harsh chemicals or solvents; 2) the antibody targeting efficiency was approximately three‐fold of EDC‐NHS conjugation; and 3) it can be used to coat a variety of functional nanoparticles regardless of their size, shape, and chemical composition.

## 2 Results and Discussion

### 2.1 Assembly of ZIF8‐Ab Coating with Favorable Ab Orientations

**Figure 2:**
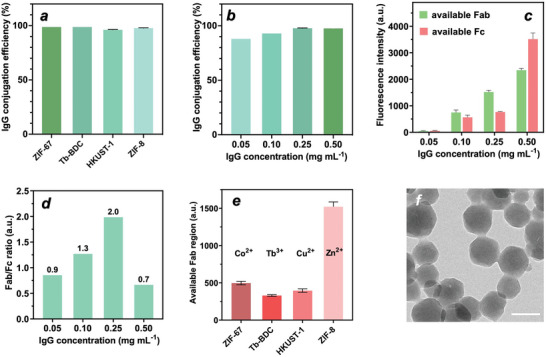
Evaluation of MOF‐antibody assembly. a) IgG conjugation efficiency of MOF@MOF‐IgG at the IgG concentration of 0.25 mg mL^−1^. b) IgG conjugation efficiency of ZIF8@ZIF8‐IgG at different concentrations of IgG (0.05–0.5 mg mL^−1^). c) Fluorescence intensity of available Fab and available Fc regions of ZIF8@ZIF8‐IgG at different concentrations of IgG (mean ± SD, *n* = 2) and d) corresponding fluorescence ratio. e) Fluorescence intensity of available Fab regions of ZIF8@ZIF8‐IgG, ZIF‐67@ZIF‐67‐IgG, Tb‐BDC@Tb‐BDC‐IgG, and HKUST‐1@HKUST‐1‐IgG (mean ± SD, *n* = 2). f) TEM image of ZIF8@ZIF8‐IgG (scale bar = 100 nm)

We first immobilized human IgG using different MOF materials containing Zn, Co, Cu, or Tb (i.e., Zn‐based ZIF‐8, Co‐based ZIF‐67, Cu‐based HKUST‐1, and Tb‐based Tb‐BDC) to determine which metal ions have a high specificity for Fc regions of antibodies. In general, the coatings were synthesized directly after mixing the metal salt, ligand, antibody of different concentrations, and presynthesized MOF core in water at 37 °C. The IgG conjugation efficiency of the MOF coatings with different metals was quantified via fluorescence spectrometry using FITC‐labeled IgG. Regardless of the MOF type, the conjugation efficiency of all MOF@MOF‐IgG increased slightly when the antibody concentration increased from 0.05 to 0.5 mg mL ^−1^ (Figure S1a–d, Supporting Information), and did not show obvious difference between different MOF coatings at the same IgG concentration (**Figure** **2**a; Figure S1a–d, Supporting Information). Specifically, ZIF‐8, ZIF‐67, Tb‐BDC, and HKUST‐1 exhibited high IgG conjugation efficiencies ranging from 96.2% to 98.8% at the IgG concentration of 0.25 mg mL^−1^, suggesting that all of these MOF materials immobilized IgG efficiently (Figure 2b; Figure S1a–d, Supporting Information). To examine how the antibody concentration influenced the antibody orientation, secondary antibodies were used to specifically bind to the available Fab regions (FITC‐labeled anti‐human IgG (Fab‐specific) antibody) and Fc regions (Cy3‐labeled anti‐human IgG (Fc‐specific)) of MOF@MOF‐IgG prepared using different antibody concentrations. The results revealed that coatings prepared from the IgG concentration of 0.25 mg mL^−1^ on ZIF‐8 had a highest fluorescence ratio of FITC to Cy3 than coatings prepared from the other IgG concentrations or MOFs (Figure 2c–d; Figure S1e–l, Supporting Information), indicating more accessible and functional Fab regions at the IgG concentration of 0.25 mg mL^−1^ on ZIF‐8 or in other words, higher antibody orientation. Such a high ratio of accessible Fab to Fc after immobilization was also confirmed via fluorescence microscopy (Figure S2a, Supporting Information). To further examine different MOF coatings in terms of accessible Fab regions, the different coatings were assembled using human IgG, followed by the addition of a secondary FITC‐labeled Fab‐specific anti‐human IgG and measurement using flow cytometry. The results demonstrated that the metal type played an important role in antibody orientation that makes Fab regions freely accessible to the secondary antibodies, where ZIF‐8 (Zn^2+^) > ZIF‐67 (Co^2+^) > HKUST‐1 (Cu^2+^) > Tb‐BDC (Tb^3+^) (Figure 2e). Specifically, the Zn‐based antibody coatings made with ZIF‐8 had at least 3‐fold increase in available Fab compared to the MOFs based on Co^2+^, Tb^3+^, and Cu^2+^, respectively. This is likely because Zn^2+^ preferentially interacts with the histidine‐rich Fc region, in which histidine essentially has a 2‐methylimidazolside group, the same organic molecule used in ZIF‐8 construction. Therefore, we restricted our further investigation to ZIF8‐Ab coatings.

The ZIF8‐IgG coatings were specifically made by adding 2.5 × 10^−3^ m Zn(NO_3_)_2_·6H_2_O to a solution of nano‐scaled ZIF‐8 core particles containing 2‐methylimidazol (Hmim, 0.25 m) and IgG (0.25 mg mL^−1^). Both ZIF‐8 and ZIF8@ZIF8‐IgG particles had a truncated cubic shape with a similar particle size of ≈100 nm on average, as observed using transmission electron microscopy and scanning electron microscopy (Figure 2f; Figure S3c,d, Supporting Information). Moreover, after the ZIF8‐IgG coating, the zeta potential of the ZIF‐8 nanoparticles decreased from +21.8 to +5.1 mV, which in conjunction with the fluorescence intensity data (Figure 2a) indicated the successful conjugation of antibodies to the surface of the nanoparticle core. Notably, without antibodies, the low concentration of 2.5 × 10^−3^ m Zn^2+^ and 0.25 m Hmim which were used to form the coating did not lead to the formation of ZIF‐8 nanoparticles (Figure S3a,b, Supporting Information). Alternatively, when using the higher concentrations of 25 × 10^−3^ m Zn(NO_3_)_2_·6H_2_O and 2.5 m Hmim (typical concentrations to prepare ZIF‐8 nanoparticles) to form the coatings in the presence of antibodies, nonselective conjugation of the antibodies was observed (Figure S3e, Supporting Information), which confirmed the importance of both the presence of antibodies and the concentration of the precursors to produce coatings of oriented antibodies.

**Figure 3:**
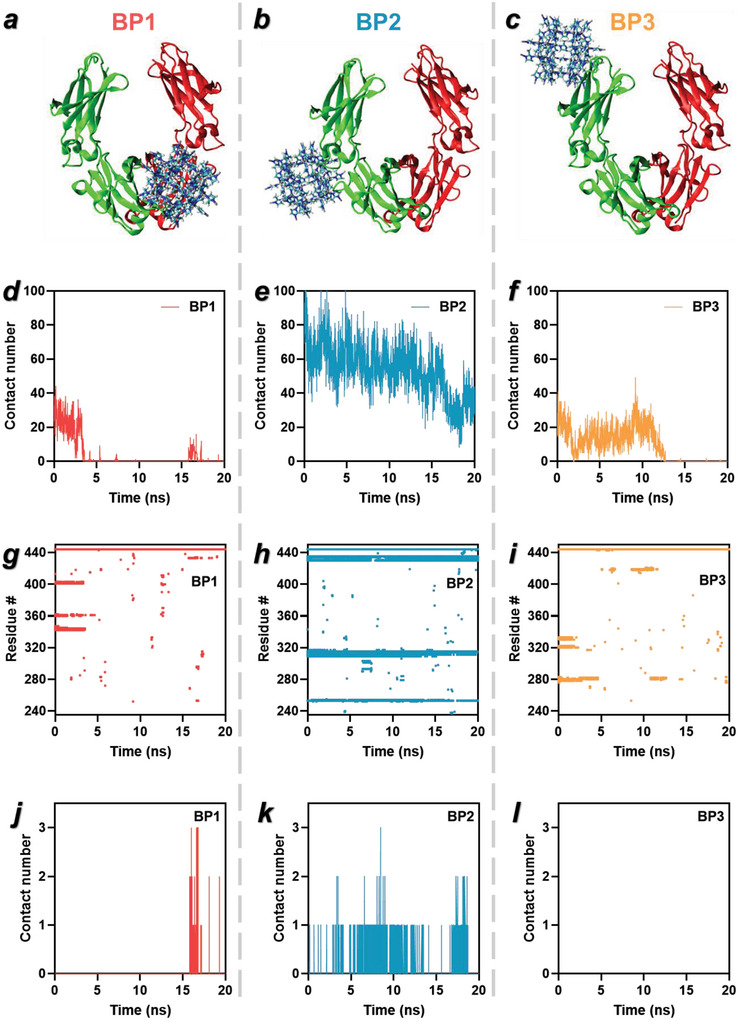
MD simulations of ZIF‐8 interacting with Fc dimers. a–c) Molecular models of three binding positions (BP1, BP2, BP3). IgG Fc dimer is colored in red and green. For ZIF‐8, carbon atoms are colored in cyan, hydrogen in white, nitrogen in blue, and zinc in gray. d–f) Time evolution of the contact number between Fc dimer and ZIF‐8. g–i) Residues of the protein contacting ZIF‐8. j–l) Time evolution of the contact number between histidine and zinc atom.

To better understand the interactions between the histidine‐rich Fc regions of antibodies and Zn‐based ZIF‐8 complexes, molecular dynamics (MD) simulations were conducted using the GROMACS software package. Firstly, an Fc dimer was used to study specific interactions between IgG Fc and ZIF‐8, and a ZIF‐8 complex was placed at six different uncontacted positions to identify the preferred binding sites of ZIF‐8 (Figure S4a, Supporting Information) and examined using 10 ns MD simulations in vacuum. As a result, we obtained three binding positions (BPs), designated as BP1, BP2, and BP3 (**Figure** **3**a–c). These three binding positions were each further solvated with TIP3 water molecules and 150 × 10^−3^ m NaCl and run for 20 ns of unbiased MD simulation to evaluate the binding stability. It was found that the BP2 position had the highest binding energy than the other two binding positions (BP1, BP3) (Figure S4b–d, Supporting Information). Further analysis revealed that ZIF‐8 had a much higher and consistent number of contacts with BP2 than with the other two binding positions (Figure 3d–f). The results indicate the strong, consistent, and specific interaction of ZIF‐8 with BP2. The specific contact residues (residues #310, 433, and 435) of BP2 were identified as different protonation states of histidine, again suggesting that interactions between zinc atoms and histidine govern the formation of the ZIF8‐Ab coatings with the Fab regions oriented out from the MOF core. Meanwhile, in the cases of BP1 and BP3, there was no consistent contact between specific residues and ZIF‐8 over the simulation period (Figure 3g–i), likely due to the lack of histidine in BP1 and BP3. To further investigate the affinity between the histidine residues in the Fc dimer and the zinc atoms of ZIF‐8, the number of contacts was calculated to be 0–3 during a 20 ns simulation, from which it was found that ZIF‐8 had nearly continuous contact with BP2, but minimal contact with BP1 and BP3 (Figure 3j–l). The Fc dimer root‐mean‐square deviation (RMSD) profiles after the addition of ZIF‐8 demonstrated that there was no obvious change of distance over 20 ns, indicating that the addition of ZIF‐8 in proximity to free Fc dimers did not change IgG Fc configuration (Figure S4e–g, Supporting Information). Overall, the MD simulation results suggested that ZIF‐8 has a strong binding affinity to the Fc region of antibodies via specifically interacting with histidine residues via zinc atoms.

### 2.2 Cell Targeting of ZIF8@ZIF8‐Ab

**Figure 4:**
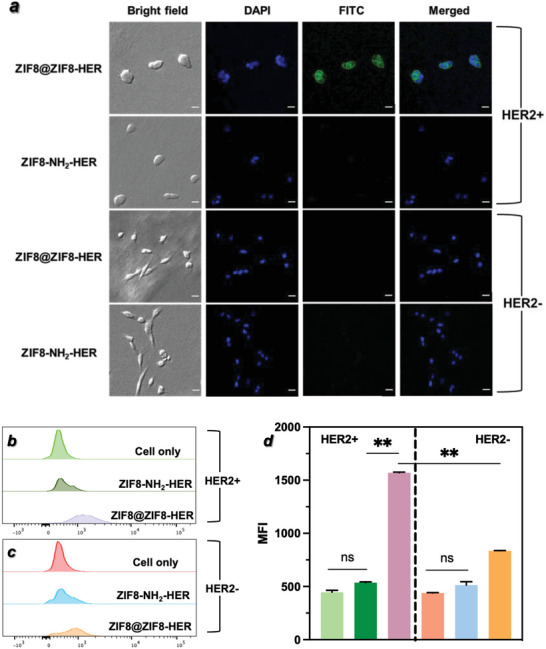
Cell targeting of ZIF8@ZIF8‐HER in BT‐474 (HER2 positive) and MDA‐MB‐231 (HER2 negative) cells, in comparison to ZIF8‐NH_2_‐HER‐FITC. a) Fluorescence microscopy images (scale bar = 20 µm). b,c) Flow cytometry histograms and d) corresponding median fluorescence intensity (MFI) analysis (mean ± SD, *n* = 2). ns: no significance difference; **p < 0.01.

The ability of ZIF8@ZIF8‐Ab to target specific cells was investigated using Herceptin (HER) as a model antibody. Herceptin is a monoclonal, FDA‐approved therapeutic antibody that specifically targets receptor tyrosine kinase human epidermal growth factor receptor 2 (HER2), which is overexpressed in various cancer cells. BT‐474 breast cancer cells were chosen as a HER2‐positive cell line as they overexpress HER2, while MDA‐MB‐231 cells were used as a negative control as they are triple‐negative breast cancer cells with very low HER2 expression levels. The cells were treated with FITC‐labeled ZIF8@ZIF8‐HER for 1 h at 37 °C, followed by washing and subsequent fluorescence analysis. In the fluorescence microscopy images ( **Figure** **4**a), the ZIF8@ZIF8‐HER‐FITC‐treated BT‐474 cells showed strong fluorescence, while minimal fluorescence was observed in ZIF8@ZIF8‐HER‐FITC‐treated MDA‐MB‐231 cells, indicating the selective targeting ability of ZIF8@ZIF8‐HER. Control FITC‐ZIF8 without Herceptin showed no significant difference compared with the untreated group in both cell lines (Figure S5a‐c, Supporting Information), highlighting that the anti‐HER2 antibody played an important role in cell recognition. Moreover, neither isotype control of FITC labelled ZIF8@ZIF8‐IgG1 which lacks specificity to the HER2 target, nor pretreatment of free Herceptin (200 µg mL^−1^ for 1 h incubation) showed significant difference compared to the untreated BT‐474 cell cultures (Figure S5d–f, Supporting Information). The results from both control experiments confirmed the specific targeting role of the anti‐HER2 antibody. The targeting ability of ZIF8@ZIF8‐HER‐FITC was then quantitatively confirmed by flow cytometry, where the median fluorescence intensity (MFI) of BT‐474 cells treated with ZIF8@ZIF8‐HER‐FITC was significantly higher (*p* < 0.01) than their MDA‐MB‐231 counterparts (Figure 4b–d).

Next, the cell targeting ability of ZIF8@ZIF8‐HER was compared with the ZIF8‐NH_2_‐HER conjugate that was prepared via traditional EDC‐NHS coupling. ZIF8‐NH_2_‐Ab was prepared by functionalizing ZIF‐8 with amine groups using 3‐amino‐1,2,4‐triazole (Atz) followed by antibody conjugation with EDC‐NHS.^[^ ^17^ ^]^ The ^1^H NMR spectra of ZIF‐8‐NH_2_ showed 81.2% Atz conversion (Figure S6a, Supporting Information), showing a large amount of available amine groups for conjugation. The conjugation efficiency of IgG to ZIF‐8‐NH_2_ was 84.0% while the fluorescence intensity ratio of Fab/Fc was 0.91 (Figure S6b, Supporting Information), which were lower than those of ZIF8@ZIF8‐IgG (97.8% and 2.0 respectively). Moreover, the fluorescence intensity ratio of Fab/Fc being ≈1 for ZIF8‐NH_2_‐IgG confirmed that the antibody orientation was completely random as Fab and Fc regions equally (i.e., nonselectively) bound to ZIF‐8 via EDC‐NHS conjugation. In comparison, our new site‐specific coating strategy showed improved antibody conjugation efficiency and higher fluorescence intensity ratio of Fab/Fc, which is superior to traditional antibody conjugation methods. In the cell targeting study, neither BT‐474 nor MDA‐MB‐231 showed significant change in fluorescence intensity after incubation with FITC‐labeled ZIF8‐NH_2_‐HER (Figure 4b–d). This can be explained by the lower level of Fab activity in ZIF8‐NH_2_‐HER. Compared to ZIF8‐NH_2_‐HER, ZIF8@ZIF8‐HER presented a remarkably higher cell binding efficiency in HER2‐overexpressed BT‐474 cells, as shown by both fluorescence microscopy and flow cytometry (Figure 4). The average MFI value of BT‐474 cells treated with ZIF8@ZIF8‐HER‐FITC was 2.9‐fold of their EDC‐NHS counterparts (Figure 4d).

### 2.3 Coating Functional Nanoparticles with ZIF8‐Ab

To validate the potential biomedical applications of ZIF8‐Ab coatings for phototherapy, drug delivery, and bioimaging, biofunctional nanoparticles were coated with ZIF8‐IgG. Four types of nanoparticles, i.e., gold nanorods (AuNR), layered double hydroxide nanosheets (LDH), superparamagnetic iron oxide nanoparticles (SPION), and ultrasmall superparamagnetic iron oxide nanoparticles (USPIO), were selected for proof‐of‐concept biomedical studies, based on their different size, shape, chemical composition, and functionality. The presynthesized nanoparticles were then coated with ZIF8‐IgG and compared against nanoparticles functionalized with EDC‐NHS in terms of antibody orientation.

**Figure 5:**
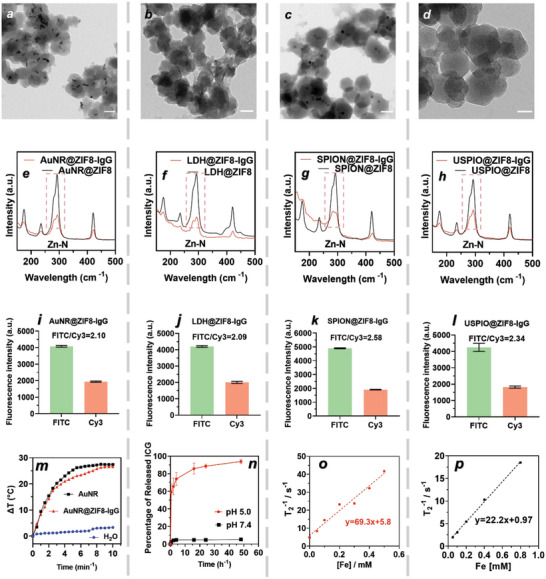
Coating functional nanoparticles with ZIF8‐Ab. TEM images of a) AuNR@ZIF8‐IgG (scale bar = 100 nm), b) LDH@ZIF8‐IgG (scale bar = 100 nm), c) SPION@ZIF8‐IgG (scale bar = 100 nm), and d) USPIO@ZIF8‐IgG (scale bar = 50 nm). THz‐FIR spectroscopy of e) AuNR@ZIF8‐IgG and AuNR@ZIF8, f) LDH@ZIF8‐IgG and LDH@ZIF8, g) SPION@ZIF8‐IgG and SPION@ZIF8, and h) USPIO@ZIF8‐IgG and USPIO@ZIF8. Median fluorescence intensity (MFI) from flow cytometry measurements of i) AuNR@ZIF8‐IgG, j) LDH@ZIF8‐IgG, k) SPION@ZIF8‐IgG, and l) USPIO@ZIF8‐IgG, showing the available Fab and Fc regions. m) Time‐dependent temperature increment of AuNR and AuNR@ZIF8‐IgG suspension ([Au] = 40 µg mL^−1^) irradiated by an 808 nm laser at a power density of 1.0 W cm^−2^. n) Drug release curves representing the percentage of ICG released in regards to the total loading ICG versus time of LDH@ZIF8‐IgG incubated at 37 °C in pH 5.0 and pH 7.4 buffer solutions. Plot of T_2_^−1^ versus Fe concentration of o) SPION@ZIF8‐IgG and p) USPIO@ZIF8‐IgG after incubation with pH 5 buffer solution at 37 °C for 24 h.

AuNR are plasmonic nanoparticles with excellent photothermal performance to treat tumors and other diseases.^[^ ^18^ ^]^ We synthesized rod‐shaped AuNR with an average length and width of 39.6 and 9.8 nm, respectively (Figure S7a, Supporting Information), via a seed mediated growth method and subsequent surface modification with poly(sodium‐4‐styrenesulfonate) (PSS). After ZIF8‐IgG coating, the AuNR@ZIF8‐IgG exhibited a core–shell structure with an average particle size of 102 ± 12 nm (**Figure** **5**a; Figure S8a, Supporting Information), nearly identical to the size of the core–shell ZIF8@ZIF8‐IgG particles. Moreover, the XRD pattern of the AuNR@ZIF8‐IgG confirmed the successful coating of ZIF8‐IgG (Figure S9a,b, Supporting Information).^[^ ^19^ ^]^ In addition, the Zn–N bond originating from the flexible ZnN_4_ tetrahedra in ZIF‐8 with and without the presence of IgG was characterized by synchrotron far‐infrared (FIR)‐terahertz (THz) radiation, showing the presence of IgG led to a blue shift of the Zn–N stretching peaks at 281 and 292 cm^−1^ by 1 cm^−1^ in AuNR@ZIF8‐IgG (Figure 5e; Figures S10 and S11a, Supporting Information).^[^ ^20^ ^]^ The THz‐FIR results indicated the Zn–N bond strength enhancement in presence of antibody and confirmed specific interactions between histidine in IgG Fc and Zn in ZIF‐8, which is consistent with the MD simulation data. To demonstrate IgG immobilization efficiency and investigate IgG orientation of AuNR@ZIF8‐IgG, FITC‐labeled anti‐human IgG (Fab‐specific) and Cy3‐labeled anti‐human IgG (Fc‐specific) were used as secondary antibodies to detect accessible Fab and Fc regions, respectively. Fluorescence microscopy images of AuNR@ZIF8‐IgG showed that the fluorescence intensity of accessible Fab was obviously higher than that of accessible Fc (Figure S2b, Supporting Information). Specifically, the fluorescence intensity ratio of Fab/Fc of AuNR@ZIF8‐IgG was measured to be 2.10 via flow cytometry (Figure 5i), suggesting that ZIF8‐coated AuNR preserved the bioavailability of Fab regions. Moreover, AuNR@ZIF8‐IgG nanoparticles had a much higher fluorescence intensity ratio of Fab/Fc than the randomly oriented AuNR@ZIF8‐NH_2_‐IgG (fluorescence intensity ratio of Fab/Fc = 1.25) (Figures S12a and S13a, Supporting Information). To evaluate the photothermal properties of coated and uncoated AuNR, the temperature change of both AuNR@ZIF8‐IgG and AuNR suspensions containing 40 µg mL^−1^ Au were recorded under 1.0 W cm^−2^ irradiation with an 808 nm laser. The temperature of AuNR@ZIF8‐IgG solution increased by 14.4 °C within 2 min irradiation and increased by 27 °C after a total of 10 min irradiation, which was similar to that of uncoated AuNR that increased by 15.4 °C within 2 min and 28 °C after 10 min irradiation (Figure 5m). Moreover, the photothermal properties of both AuNR@ZIF8‐IgG remained unchanged after five cycles of heating and cooling (Figure S14a, Supporting Information), which was similar to that of AuNR and indicated the desirable photo‐stability of AuNR@ZIF8‐IgG. These results demonstrated that the photothermal effect of AuNR remains unchanged after ZIF8‐IgG coating, confirmig that ZIF8‐IgG does not inhibit or interfere with the inherent properties of AuNR and thereby preserves their potential for photothermal treatment. In addition, the effect of increased temperature on antibody activity was studied by evaluating accessible Fab and Fc regions using secondary antibodies (FITC‐labeled anti‐human IgG (Fab‐specific) antibody and Cy3‐labeled anti‐human IgG (Fc‐specific) antibody) before and after heating ZIF8@ZIF8‐IgG for 10 min at 50 °C. The results showed that the fluorescence ratio of FITC to Cy3 remained unchanged after heating (Figure S14b,c, Supporting Information), indicating that increased temperature (up to 50 °C) had a neglectable effect on antibody activity.

LDH nanosheets were fabricated via a co‐precipitation method, followed by room temperature aging and POEGA‐*b*‐PMAEP functionalization. The LDH nanosheets exhibited a 2D disc‐like morphology with an average size of 52 ± 9 nm and a thickness of 11.3 nm (Figures S7b, S15 and Table S1, Supporting Information). The ZIF8‐IgG‐coated LDH (LDH@ZIF8‐IgG) had an average particle size of 147 nm (Figure 5b; Figure S8b, Supporting Information). Notably, LDH has much lower contrast compared to ZIF‐8 under TEM, however LDH on its side could be observed as a needle‐like morphology.^[^ ^21^ ^]^ Similar THz‐FIR data were obtained when comparing LDH@ZIF8‐IgG to LDH@ZIF8, which showed that the presence of IgG led to a blue shift of the Zn–N stretching peaks at 281 and 292 cm^−1^ by 1 cm^−1^ (Figure 5f; Figures S10 and S11b, Supporting Information), demonstrating the enhanced Zn–N bond strength and interactions between histidine‐rich IgG Fc and Zn‐containing ZIF‐8. The fluorescence intensity ratio of Fab/Fc of LDH@ZIF8‐IgG was 2.09, which was much higher than LDH@ZIF8‐NH_2_‐IgG with a ratio of 1.4 (Figure 5j; Figure S2c, S12b and S13b, Supporting Information). The results demonstrated that IgG can be favorably orientated on LDH with ZIF‐8 as a coating. To evaluate the possibility for drug delivery, indocyanine green (ICG) was loaded onto the LDH nanosheets, followed by ZIF8‐IgG coating, yielding LDH‐ICG@ZIF8‐IgG. The ICG loading efficiency was 91.7%, confirming successful drug encapsulation. The XRD patterns of LDH‐ICG@ZIF8‐IgG also confirmed the successful coating of ZIF8‐IgG (Figure S9c,d, Supporting Information).^[^ ^19^, ^22^ ^]^ The drug was released in an acetate buffer solution of pH 5.0, which mimics intracellular pH value, while PBS of pH 7.4 was used as a control to mimic the standard biological pH environment of blood circulation. LDH‐ICG@ZIF8‐IgG showed a prolonged release profile at pH 5.0 with 93% ICG released during a 48 h incubation period, whereas only 5% of ICG released at pH 7.4 at the same time period (Figure 5n). All these data demonstrate that the ZIF8‐IgG can modulate the drug release of the encapsulated cargo, which highlights the promise of ZIF8‐IgG coatings for drug delivery applications.

Iron oxide nanoparticles have been extensively explored for their use in enhancing magnetic resonance imaging (MRI). Iron oxide nanoparticles (SPION and USPIO) were coated with ZIF8‐IgG, and the resultant SPION@ZIF8‐IgG and USPIO@ZIF8‐IgG were then evaluated for antibody orientation and MRI performance. The SPION and USPIO nanoparticles were spherical with an average size of 25 and 3 nm, respectively, as observed under TEM, and the SPION@ZIF8‐IgG and USPIO@ZIF8‐IgG showed average particle sizes of 109 and 100 nm, respectively (Figures S7c,d and S8c,d, Supporting Information; Figure 5c,d). Furthermore, the XRD patterns of ZIF8‐IgG coated SPION and USPIO nanoparticles confirmed the presence of ZIF‐8 (Figure S9e–h, Supporting Information).^[^ ^19^, ^23^ ^]^ The iron oxide nanoparticles were homogenously distributed in USPIO@ZIF8‐IgG, which can be important for maintaining the MRI performance. THz‐FIR also confirmed the blue shift of IgG (Figure 5g,h; Figures S10 and S11c,d, Supporting Information), demonstrating that the specific interaction between IgG Fc and ZIF‐8. The fluorescence intensity ratio of Fab/Fc of SPION@ZIF8‐IgG and USPIO@ZIF8‐IgG were 2.58 and 2.34 respectively, which were twice as high as that of SPION@ZIF8‐NH_2_‐IgG and USPIO@ZIF8‐NH_2_‐IgG (Figure 5k,l; Figures S2d,e, S12c,d, and S13c,d, Supporting Information). Moreover, the total amount of accessible Fab in SPION@ZIF8‐IgG and USPIO@ZIF8‐IgG were three times higher than their EDC‐NHS counterparts, as evidenced by the FITC fluorescence intensity. Next, the MRI performance of the iron oxide nanoparticle with or without ZIF8‐IgG coating was evaluated in buffer mimicking the intercellular environment. Both SPION@ZIF8‐IgG and USPIO@ZIF8‐IgG exhibited promising T_2_‐MRI performance. Although the T_2_‐weighted relaxivities of SPION@ZIF8‐IgG (*r*_2_ = 69.3 mm ^−1^ s^−1^) and USPIO@ZIF8‐IgG (*r*_2_ = 22.2 mm ^−1^ s^−1^) (Figure 5o,p; Figure S16a–c, Supporting Information) were lower than their uncoated counterparts, they are comparable to commercial T_2_‐MRI contrast agent (Combidex, *r*_2_ = 65 mm ^−1^ s^−1^).^[^ ^24^ ^]^

## 3 Conclusions

In summary, an effective site‐specific antibody conjugation strategy was established via a facile film‐coating method to improve antibody orientation for enhanced cell targeting. Due to the specific interaction between Zn in ZIF‐8 and the histidine‐rich and highly conserved Fc regions on antibodies, ZIF‐8 can simultaneously embed and favorably orientate antibodies to expose the functional Fab regions to their biological target. As a result, this coating technique generated significantly higher fluorescence intensity ratio of Fab/Fc and cell targeting efficiency than the conventional chemical conjugation method using EDC‐NHS coupling. Further application of this coating strategy to immobilize antibodies on functional nanoparticles demonstrated its potentials in drug delivery, phototherapy, and bio‐imaging. We envision that this platform nanotechnology will be a powerful tool to advance targeted biotechnology applications.

## 4 Experimental Section

### Materials

2‐methylimidazole (Hmim, 99%), Zn(NO_3_)_2_·6H_2_O (>98%), TbCl_3_∙6H_2_O (99.9%), Cu(NO_3_)_2_·3H_2_O (99%), Co(NO_3_)_2_∙6H_2_O (98%), ferric acetylacetonate (Fe(acac)_3_), diphenyl ether, oleic acid, oleylamine, 1‐octadecene, 1‐octadecanol, cyclohexane, acetone, and tetrahydrofuran (THF), PVP (polyvinylpyrrolidone, average MW 8000), sodium borohydride (NaBH_4_), cetyltrimethylammonium bromide (CTAB), hydrogen tetrachloroaurate (III) trihydrate (HAuCl_4_·3H_2_O), silver nitrate (AgNO_3_), l‐ascorbic acid (AA), sodium sulfate (Na_2_SO_4_), poly(sodium‐4‐styrenesulfonate) (PSS, MW 70 000), MgCl_2_·6H_2_O (>99.0%), AlCl_3_·6H_2_O (>99.0%), NaOH (>98%), trimesic acid (H_3_BTC, 95%), disodium terephthalate (Na_2_BDC, 99+ %), 3‐amino‐1,2,4‐triazole (Atz, >95%), *N*‐(3‐dimethylaminopropyl)‐*N*′‐ethylcarbodiimide hydrochloride (EDC, 98%), *N*‐hydroxysuccinimide (NHS), IgG1, Kappa from human myeloma plasma (I5154), human IgG (purified immunoglobulin, Reagent Grade), IgG‐FITC from human serum (F9636), anti‐human IgG (Fab‐specific)‐FITC produced in goat, anti‐human IgG (Fc‐specific) Cy3 conjugate developed in goat and fluorescein isothiocyanate isomer (FITC, >90%) were purchased from Sigma‐Aldrich. Deuterium oxide (D_2_O, 99.9 at% D) was purchased from Cambridge Isotope Laboratories, Inc. Indocyanine green (ICG) was bought from sapphire biosciences. Iron oxide nanoparticles (25 nm) were provided by Imagion Biosystems, Inc. PEG derivatives (MW ≈ 2000) with diphosphate (DP) were customized products provided by Suzhou Xinying Biomedical Technology Co., Ltd., Agarose were purchased from BIO‐RED. Herceptin (trastuzumab) was purchased from Roche Australia. Dulbecco's Modified Eagle's Medium (DMEM, high glucose), Roswell Park Memorial Institute medium (RPMI 1640) and penicillin/streptomycin were bought from Gibco. Fetal bovine serum (FBS) and ProLong Gold Antifade Mountant with DAPI were purchased from Thermo Fisher Scientific. Trypsin‐EDTA (0.25%, phenol red) was purchased from Life Technologies Australia. Cell counting kit‐8 (CCK‐8) was bought from Abcam. Phosphonic acid terminated poly(ethylene glycol) (POEGA‐*b*‐PMAEP) was synthesized using a controlled/living radical polymerization according to procedures described previously.^[^ ^25^ ^]^ Milli‐Q water and nitrogen gas were used from our own laboratory.

### Assembling Different MOF‐Ab Coatings

ZIF‐8 nanoparticles were synthesized by mixing aqueous solutions of Hmim (205 mg, 2.5 m) and Zn(NO_3_)_2_·6H_2_O (7.44 mg, 25 × 10^−3^ m) and shaking for 30 min. After centrifugation and washing (6000 rpm, 5 min), ZIF‐8 nanoparticles were obtained.

To assemble the ZIF8‐Ab coatings on ZIF‐8 (ZIF8@ZIF8‐IgG), the ZIF‐8 suspension was added to Hmim (20.5 mg, 0.25 m) containing 0.05, 0.25, 0.1, 0.5 mg mL^−1^ human IgG respectively, then mixed with Zn(NO_3_)_2_·6H_2_O (0.744 mg, 2.5 × 10^−3^ m), followed by shaking for 30 min at 37 °C, and subsequent centrifugation (6000 rpm, 5 min) and washing steps.

To prepare ZIF‐67@ZIF‐67‐IgG, ZIF‐67 was first synthesized by mixing Co(NO_3_)_2_∙6H_2_O solution (4.57 mg, 25 × 10^−3^ m) and Hmim (205 mg, 2.5 m) with shaking at 37 °C for 30 min, followed by centrifugation and washing (6000 rpm, 5 min). The ZIF‐67 suspension was then mixed with Hmim (205 mg, 2.5 m), Co(NO_3_)_2_·6H_2_O solution (4.57 mg, 25 × 10^−3^ m), and 0.05, 0.25, 0.1, 0.5 mg mL^−1^ human IgG respectively, followed by shaking at 37 °C for 30 min. Then the particles were centrifuged and washed to obtain ZIF‐67@ZIF‐67‐IgG.

To prepare Tb‐BDC@Tb‐BDC‐IgG, Tb‐BDC was first synthesized by mixing 20 × 10^−3^ m Na_2_BDC and 20 × 10^−3^ m TbCl_3_∙6H_2_O with shaking at 37 °C for 30 min, followed by centrifugation (6000 rpm, 5 min). Tb‐BDC suspension was then mixed with 20 × 10^−3^ m Na_2_BDC, 20 × 10^−3^ m TbCl_3_·6H_2_O and 0.05, 0.25, 0.1, 0.5 mg mL^−1^ human IgG respectively, followed by shaking at 37 °C for 30 min, centrifugation and washing cycles (6000 rpm, 5 min) to obtain Tb‐BDC@Tb‐BDC‐IgG.

To fabricate HKUST‐1@HKUST‐1‐IgG, HKUST‐1 was first prepared by mixing an aqueous suspension of H_3_BTC dissolved in 1:1 water/ethanol mixture, and an aqueous solution of Cu(NO_3_)_2_∙3H_2_O to a final molar ratio of Cu:H_3_BTC = 1:2 in 1 mL solution with shaking at 37 °C for 1 h, collected by centrifugation (6000 rpm, 5 min). The HKUST‐1 suspension was then mixed with H_3_BTC suspension, Cu(NO_3_)_2_∙3H_2_O at the same molar ratio, and 0.05, 0.25, 0.1, 0.5 mg mL^−1^ human IgG respectively, followed by shaking at 37 °C for 1 h, centrifugation and washing cycles (6000 rpm, 5 min) to obtain HKUST‐1@HKUST‐1‐IgG.

### Surface Functionalization of Antibodies on ZIF‐8 Nanoparticles via EDC‐NHS Coupling

3‐amino‐1,2,4‐triazole (Atz, 48 mg, 0.56 mmol) was added to the ZIF‐8 suspension (5 mL, 10 mg mL^−1^) and coupling was performed at 50 °C for 2 h.^[^ ^26^ ^]^ The product was collected after 2 cycles of centrifugation at 6000 rpm for 5 min. The obtained ZIF8‐NH_2_ was suspended in 1 mL water. The EDC‐NHS reaction was then conducted at pH 7.4 and 37 °C. Human IgG (0.25 mg) was incubated with EDC (5.1 mg, 100 µL) for 30 min, followed by incubation with NHS (7.7 mg, 100 µL) for another 30 min to obtain NHS‐IgG. ZIF8‐NH_2_ was then added to NHS‐IgG and incubated for 2 h. The product was centrifuged and redispersed in 1 mL water.

### Evaluation of Antibody Conjugation Efficiency and Antibody Conjugation Capacity

Fluorescent IgG‐FITC was used to assemble antibody‐nanoparticle conjugates by following the aforementioned procedures used to synthesize nonfluorescent IgG‐nanoparticle conjugates. After centrifugation and washing (6000 rpm, 5 min), the supernatant was collected and measured via fluorescence spectrometry at *λ*_ex_ = 495 nm and *λ*_em_ = 519 nm. The IgG conjugation efficiency and capacity were calculated by following equations

(1) $$\begin{array}{ccc} & & \mathrm{IgG}\;\;\mathrm{conjugation}\;\;\mathrm{efficiency}\;\;\left(\%\right)=(\mathrm{mass}\;\;\mathrm{of}\;\;\mathrm{IgG}\;\;\mathrm{added}\\ & & \;-\;\mathrm{mass}\;\;\mathrm{of}\;\;\mathrm{IgG}\;\;\mathrm{in}\;\;\mathrm{supernatant})/\left(\mathrm{mass}\;\;\mathrm{of}\;\;\mathrm{IgG}\;\;\mathrm{added}\right)\times 100\% \end{array}$$

(2) $$\begin{array}{ccc} & & \mathrm{IgG}\;\mathrm{Conjugation}\;\mathrm{Capacity}\;\;\left(\%\right)=\left(\mathrm{mass}\;\;\mathrm{of}\;\;\mathrm{IgG}\;\;\mathrm{added}\right.\\ & & \;\left.-\;\mathrm{mass}\;\;\mathrm{of}\;\;\mathrm{IgG}\;\;\mathrm{in}\;\;\mathrm{supernatant}\right)/\left(\mathrm{mass}\;\;\mathrm{of}\;\;\mathrm{MOF}\;\;\mathrm{particles}\right)\times 100\% \end{array}$$

### Evaluation of Antibody Orientation via Fluorescence Imaging

MOF‐Abs or ZIF8‐IgG coated bifunctional nanoparticles (100 µL) was incubated with both FITC‐labeled anti‐Human IgG (Fab‐specific) produced in goat (working dilution 1:1000, 10 µL) and Cy3‐labeled anti‐Human IgG (Fc‐specific) produced in goat (working dilution 1:1000, 10 µL) for 30 min at 37 °C, followed by two centrifugation/wash steps at 6000 rpm for 5 min, and finally dispersed in 100 µL water. The fluorescence intensities of FITC and Cy3 were measured by fluorescence imaging at *λ*_ex_ = 495 nm and *λ*_em_ = 519 nm for FITC and *λ*_ex_ = 552 nm and *λ*_em_ = 570 nm for Cy3.

### Quantification of Antibody Orientation via Flow Cytometry

MOF‐Abs or ZIF8‐IgG coated bifunctional nanoparticles (500 µL) was incubated with FITC‐labeled anti‐human IgG (Fab‐specific) produced in goat and Cy3‐labeled antihuman IgG (Fc‐specific) produced in goat for 30 min at 37 °C with the feeding molar ratio of IgG, IgG (Fab‐specific), and IgG (Fc‐specific) being 1:1:1. Following twice centrifugation at 6000 rpm for 5 min and dispersed in 500 µL water, the level of FITC and Cy3 fluorescence signals was measured byflow cytometry at *λ*_ex_ = 495 nm and *λ*_em_ = 519 nm for FITC and *λ*_ex_ = 552 nm and *λ*_em_ = 570 nm for Cy3.

### All‐Atom Molecular Dynamics (MD) Simulations

All simulations were performed using the GROMACS 2019 software.^[^ ^27^ ^]^ A time set up of 2 fs was used, and all simulations were run for 20 ns. The constant temperature of 310 K was maintained and periodic boundary conditions were applied. System snapshots and movies were generated by VMD.^[^ ^28^ ^]^ The ZIF‐8^[^ ^29^ ^]^ and human IgG Fc dimer (PDB ID: 7LBL) were obtained from ​​the Cambridge Crystallographic Data Centre and RCSB database, respectively. CHARMM36m all‐atom force field parameters for protein, water and ions (Na^+^, Cl^−^) were applied.^[^ ^30^ ^]^

To identify the preferred binding sites of ZIF‐8 on IgG Fc dimer, ZIF‐8 was placed at six different uncontacted positions relative to IgG Fc dimer to construct six different simulation systems in vacuum for 10 ns MD simulations. Bonded and nonbonded parameters compatible to CHARMM36m force field for ZIF‐8 were constructed according to recent force field benchmark research.^[^ ^31^ ^]^ Three binding poses were obtained, followed by further solvated with TIP3 water molecules and 150 × 10^−3^ m NaCl and run for 20 ns unbiased MD simulation each for the evaluation of the binding stability.

### Cell Culture

Breast cancer cells (BT‐474 and MDA‐MB‐231) were cultured in growth medium (RPMI1640 and DMEM with glutamine respectively) supplemented with 10% fetal bovine serum (FBS), streptomycin (100 mg mL^−1^) and penicillin (100 units mL^−1^). The cells were cultured at 37 °C in a humidified atmosphere with 5% CO_2_ in air.

### Cell Targeting Study

BT‐474 cells and MDA‐MB‐231 cells were seeded in 24‐well plates with coverslips at a density of 5 × 10^4^ cells per well. After overnight incubation, culture medium was replaced with medium containing 0.08 mg mL^−1^ FITC‐labeled ZIF8@ZIF8‐HER and 0.08 mg mL^−1^ FITC‐labeled ZIF8@ZIF8‐NH_2_‐HER. After 1 h incubation, cells were washed with PBS three times and fixed with 4% PFA, and the coverslip mounted on glass slides with ProLong Gold Antifade Mountant with DAPI. The cellular targeting efficiency of ZIF8@ZIF8‐HER and ZIF8@ZIF8‐NH_2_‐HER was observed using Olympus IX53 fluorescent microscope.

The percentage of fluorescent cells was quantified by flow cytometry. BT‐474 cells and MDA‐MB‐231 cells were seeded in 6‐well plates at a density of 3 × 10^5^ cells per well. After 24 h incubation, culture medium was replaced with medium containing 0.08 mg mL^−1^ FITC‐labeled ZIF8@ZIF8‐HER, 0.08 mg mL^−1^ FITC‐labeled ZIF8@ZIF8‐NH_2_‐HER, 0.08 mg mL^−1^ FITC‐labeled ZIF8, and 0.08 mg mL^−1^ FITC‐labeled ZIF8@ZIF8‐IgG1. After 1 h incubation, the cells were washed three times, collected and fixed with 4% PFA. In Herceptin pretreatment control cultures, the cells were seeded at the same cell density. After 24 h incubation, the cells were firstly treated with medium containing 200 µg ml^−1^ of Herceptin for 1 h. After 1 h, the medium was replaced by medium containing 0.08 mg mL^−1^ FITC‐labeled ZIF8@ZIF8‐HER and 200 µg mL^−1^ of Herceptin. After 1 h incubation, the cells were washed three times, collected and fixed with 4% PFA. The cell samples were measured using BD LSRFortessa SORP X‐20 analyzing flow cytometer. All cell experiments were carried out in duplicate, and the values from each experiment calculated from 3 wells.

### Synthesis of Biofunctional Nanoparticles

To synthesize gold nanorods,^[^ ^32^ ^]^ CTAB solution (1.0 mL, 0.20 m) was first mixed with HAuCl_4_ (1.0 mL, 0.5 × 10^−3^ m) and ice‐cold NaBH_4_ (0.12 mL, 0.01 m). The seed solution was vigorously stirred for 2 min and then kept at 25 °C for 2 h until the solution appeared brownish yellow color. The growth solution was prepared by mixing CTAB (200 mL, 0.1 m), AgNO_3_ (5.6 mL, 4 × 10^−3^ m) and HAuCl_4_ (6.5 mL, 23 × 10^−3^ m) in a 250 mL flask. Ascorbic acid (1.8 mL, 0.08 m) was dropwise added to the mixture until the solution became transparent. Finally, 1.8 mL of the seed solution was added to the mixture at 30 °C. The color of the mixture gradually changed from transparent to dark purple within 15 min. After incubating at 30 °C for 12 h, the mixture was centrifuged to remove excess CTAB surfactant. To obtain PSS coated AuNR, CTAB‐AuNR suspension (4 mL) was mixed with PSS solution (6 mL, 10 mg mL^−1^, containing 6 × 10^−3^ m NaCl) and kept for 2 h. The mixture was then centrifuged at 4000 × *g* for 10 min. The slurry was redispersed in 0.5 mL water for further use.

LDH nanosheets were prepared via a co‐precipitation method.^[^ ^33^ ^]^ A solution (5 mL) containing MgCl_2_·6H_2_O (3 × 10^−3^ m) and AlCl_3_·6H_2_O (1 × 10^−3^ m) was quickly added into NaOH solution (40 mL, 8 × 10^−3^ m) while stirring vigorously. After stirring for 20 min, the fresh MgAl‐LDH slurry was collected via centrifugation, washed twice, and re‐suspended in water (40 mL). The suspension was kept at room temperature for 72 h. After that, LDH suspension (2 mL, 4 mg mL^−1^) was sonicated for 30 s and dispersed in POEGA‐*b*‐PMAEP solution (80 µL, 0.1 g mL^−1^), followed by stirring at room temperature for 2 h. To obtain ICG loaded LDH (LDH‐ICG) nanoparticles, the PEG‐modified LDH was dispersed in ICG solution (8 mL, 1 mg mL^−1^) with stirring at room temperature for 2 h. After centrifugation (9000 × *g*, 10 min), the resultant LDH‐ICG was dispersed in water for further use.

To synthesize USPIO,^[^ ^34^ ^]^ 1.41 g (4 mmol) of Fe(acac)_3_, 3.39 g (12 mmol) of oleic acid, 3.21 g (12 mmol) of oleylamine, and 5.41 g (20 mmol) of 1‐octadecanol were dissolved in 40 mL of diphenyl ether. After being purged with nitrogen for 30 min, the solution was refluxed for 30 min under stirring. Then the reaction system was cooled to room temperature. The resultant nanoparticles were precipitated by acetone, collected by centrifugation, washed with acetone/ cyclohexane for twice, and finally redispersed in THF for further experiments. To exchange the ligand, 100 mg of PEG derivative (MW ≈ 2000) with diphosphate (DP) was dissolved in 7 mL of THF containing 10 mg hydrophobic Fe_3_O_4_ nanoparticles. Then, the reaction mixture was heated to 35 °C and kept at this temperature for 24 h under stirring. After that, the Fe_3_O_4_ nanoparticles were cooled down to room temperature and precipitated by cyclohexane, washed with THF/cyclohexane for twice, and then dried under vacuum at room temperature. The obtained hydrophilic particle powders were redispersed in water, followed by purification via ultrafiltration for 2 cycles using 100 kDa MWCO centrifugal filter (Millipore YM‐100).

### Coating ZIF8‐IgG on Biofunctional Nanoparticles

ZIF8‐IgG was assembled on bio‐functional nanoparticles by following the procedure that was used to prepare ZIF8@ZIF8‐IgG. Biofunctional nanoparticles (20 µL) were added in aqueous solutions of Hmim (205 mg, 2.5 m), and Zn(NO_3_)_2_·6H_2_O (7.44 mg, 25 × 10^−3^ m), followed by shaking for 30 min. After centrifugation and washing cycles, the particles were added in aqueous solutions of Hmim (20.5 mg, 0.25 m, containing 0.25 mg mL^−1^ human IgG), and then mixed with Zn(NO_3_)_2_·6H_2_O (0.744 mg, 2.5 × 10^−3^ m) with shaking at 37 °C for 30 min, followed by centrifugation and washing cycles.

### Effect of Heat on Antibodies

Suspensions containing ZIF8@ZIF8‐IgG nanoparticles were incubated in a shaking water bath at a speed of 200 rpm at 50 °C for 10 min. After cooling down to room temperature, ZIF8@ZIF8‐IgG nanoparticles were incubated with FITC‐labeled antihuman IgG (Fab‐specific) produced in goat and Cy3‐labeled anti‐human IgG (Fc‐specific) produced in goat for 30 min at 37 °C with the feeding molar ratio of IgG, IgG (Fab‐specific), and IgG (Fc‐specific) being 1:1:1. Following twice centrifugation at 6000 rpm for 5 min and redispersed in 500 µL water, the level of FITC and Cy3 fluorescence signals was measured by flow cytometry at *λ*_ex_ = 495 nm and *λ*_em_ = 519 nm for FITC and *λ*_ex_ = 552 nm and *λ*_em_ = 570 nm for Cy3. The fluorescence intensity was compared with the ZIF8@ZIF8‐IgG nanoparticle without heating.

### Photothermal Evaluation

Suspensions containing AuNR and AuNR@ZIF8‐IgG nanoparticles at 40 mg mL^−1^ Au concentration were placed in cuvettes for photothermal evaluation respectively. The suspension was irradiated by 808 nm NIR laser with 1.0 W cm^−2^ output density. The temperature variation of the suspension was monitored by a FLIR ONE infrared thermal camera at 30 s internal.

### Drug Release Study

LDH@ZIF8‐IgG (10 mg mL^−1^) was suspended in the HAc‐NaAc buffer of pH 5.0 and PBS buffer of pH 7.4 with a shaking speed of 300 rpm at 37 °C. At predetermined time intervals (0, 0.5, 2, 4, 16, 24, 48 h), the supernatant of aliquots was collected by centrifugation for absorbance measurement at 700 nm. The proportion of ICG released was calculated as: Released ICG percentage (%) = (mass of ICG in supernatant / mass of total ICG) × 100%.

The ICG loading efficiency and loading capacity were calculated to be

(3) $$\begin{array}{ccc} & & \mathrm{ICG}\;\;\mathrm{loading}\;\;\mathrm{efficiency}\;\;\left(\%\right)=(\mathrm{mass}\;\;\mathrm{of}\;\;\mathrm{ICG}\;\;\mathrm{added}\\ & & \;-\;\mathrm{mass}\;\;\mathrm{of}\;\;\mathrm{ICG}\;\;\mathrm{in}\;\;\mathrm{supernatant})/\left(\mathrm{mass}\;\;\mathrm{of}\;\;\mathrm{ICG}\;\;\mathrm{added}\right)\times 100\% \end{array}$$

(4) $$\begin{array}{ccc} & & \mathrm{ICG}\;\;\mathrm{loading}\;\;\mathrm{capacity}\;\;\left(\%\right)=(\mathrm{mass}\;\;\mathrm{of}\;\;\mathrm{ICG}\;\;\mathrm{added}\\ & & \;-\;\mathrm{mass}\;\;\mathrm{of}\;\;\mathrm{ICG}\;\;\mathrm{in}\;\;\mathrm{supernatant})/\left(\mathrm{mass}\;\;\mathrm{of}\;\;\mathrm{LDH}\;\;\mathrm{particles}\right)\times 100\% \end{array}$$

### Magnetic Resonance Imaging Evaluation

To evaluate MRI performance, USPIO, SPION, USPIO@ZIF8‐IgG, and SPION@ZIF8‐IgG nanoparticles were dispersed in HAc‐NaAc buffer of pH 5.0. The mixture was then diluted with the 0.5% agarose buffer solution to achieve the desired concentrations, and transferred to 2 mL Eppendorf tubes for MRI scanning. The relaxivity measurements were carried out on a 9.4T Bruker Biospec Avance III 94/20 system (Bruker, Ettlingen, Germany). A multi, spin echo sequence with 64 echos (echo spacing 10 ms) sequence was used to measure T_2_, and its parameters were TR = 10 000 ms and TE = 10.3 ms.

### Characterizations

The morphology of nanoparticles was measured using a transmission electronic microscope (TEM, FEI Tecnai G2) at an accelerating voltage of 200 kV. Zeta potential was recorded on Malvern Zetasizer Nano Series. Scanning electron microscopy (SEM) images were taken on a FEI Nova NanoSEM 230 under 10 kV acceleration voltage with a secondary electron detector. The samples were coated using Emitech K575× evaporative premium coater prior to imaging. X‐ray diffraction (XRD) measurements were performed on powder and film samples using PANalytical X‐ray diffraction system with a CoK*α* and CuK*α* source respectively. The angles were converted to CuK*α* source for comparison using HighScore Plus software. Synchrotron terahertz infrared spectroscopy (THz‐FIR) was conducted on Bruker IFS 125/HR Fourier Transform (FT) spectrometer. The fluorescence intensity of cells and nanoparticles was examined by using BD LSRFortessa SORP X‐20 analyzing flow cytometer and Olympus IX53 fluorescent microscope. ^1^H NMR spectra were acquired by dissolving the ZIF‐8 NPs in diluted HCl with D_2_O (1/99 v/v) at 25 °C on a Bruker Avance III 400 MHz NMR (Rabi). MRI measurements were carried on the 9.4 T Bruker BioSpec Avance III 94/20 system (Bruker, Ettlingen, Germany). The temperature change during the irradiation was recorded by an IR thermal camera. Flow cytometry data were analyzed and represented by Flowjo software.

### Statistical Analyses

The quantitative data were expressed as mean ± SD. Statistical differences were calculated by using two‐tailed Student's *t*‐test to compare two groups (GraphPad Prism 9.0).  *p* values less than 0.05 were considered as statistically significant differences among the compared groups, to which different asterisks were assigned (*p < 0.05; **p < 0.01; ***p < 0.001).

## Conflict of Interest

The authors declare no conflict of interest.

## Supporting information

Supporting Information

## Data Availability

The data that support the findings of this study are available from the corresponding author upon reasonable request.
