# Supplementary material for: Site‐Specific Antibody Assembly on Nanoparticles via a Versatile Coating Method for Improved Cell Targeting
Source: Adv Sci (Weinh). 2023 Jan 25;10(9):2206546. doi: 10.1002/advs.202206546 (PMC10037962; doi:10.1002/advs.202206546)
Supplement: Supplementary file 1 — Supporting Information [file ADVS-10-2206546-s001.pdf]

ADVANCED SCIENCE
Open Access

Supporting Information

for *Adv. Sci.*, DOI 10.1002/advs.202206546

Site-Specific Antibody Assembly on Nanoparticles via a Versatile Coating Method for Improved Cell Targeting

Qianyi Zhang, Jieying Liang, Andre Bongers, Joseph J. Richardson, Kang Liang and Zi Gu**

Supporting Information

Site-specific antibody assembly on nanoparticles via a versatile coating method for improved cell targeting

Qianyi Zhang, Jieying Liang, Andre Bongers, Joseph J. Richardson, Kang Liang*, Zi Gu*

Q. Zhang, Dr. J. Liang, Dr. K. Liang, Dr. Z. Gu

School of Chemical Engineering, University of New South Wales, Sydney, NSW 2052, Australia

Australian Centre for NanoMedicine (ACN), University of New South Wales, Sydney, NSW 2052, Australia

E-mails: kang.liang@unsw.edu.au ; zi.gu1@unsw.edu.au

Dr. A. Bongers

Biological Resources Imaging Laboratory, Mark wainwright Analytical Centre, The University of New South Wales, Sydney

Dr. J. J. Richardson

School of Engineering, RMIT University, Melbourne, Victoria 3000, Australia

Dr. Z. Gu

UNSW RNA Institute, University of New South Wales, NSW 2052, Australia

Dr. K. Liang

Graduate School of Biomedical Engineering, The University of New South Wales, Sydney, NSW 2052, Australia

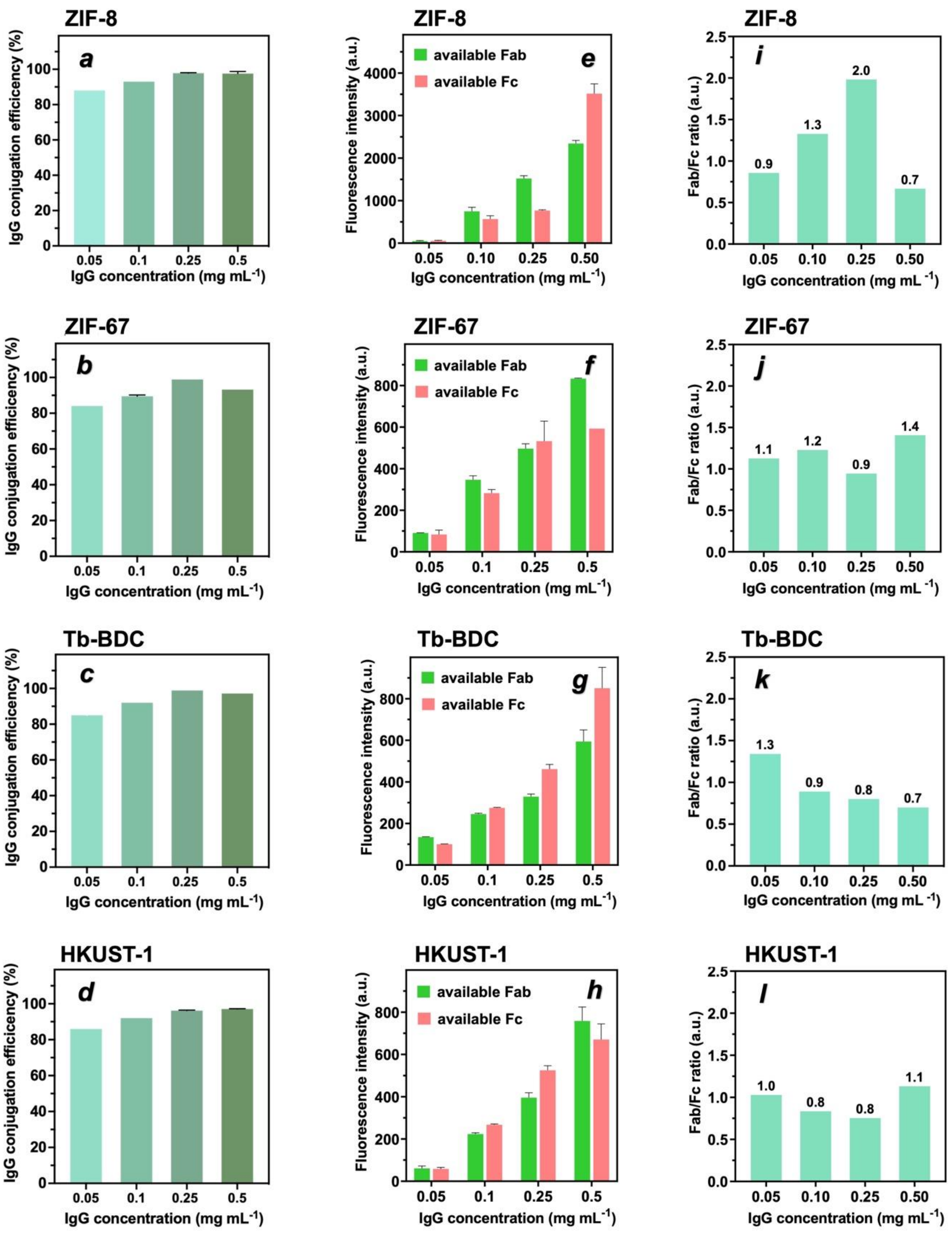

Figure S1. Evaluation of MOF-antibody assembly. (a-d) IgG conjugation efficiency of ZIF8@ZIF8-IgG at different concentrations of IgG (0.05-0.5 mg mL^{-1}). (e-h) Fluorescence intensity of available Fab and available Fc regions of MOF@MOF-IgG at different concentrations of IgG (mean ± SD, n = 2) and (i-l) corresponding fluorescence ratio.

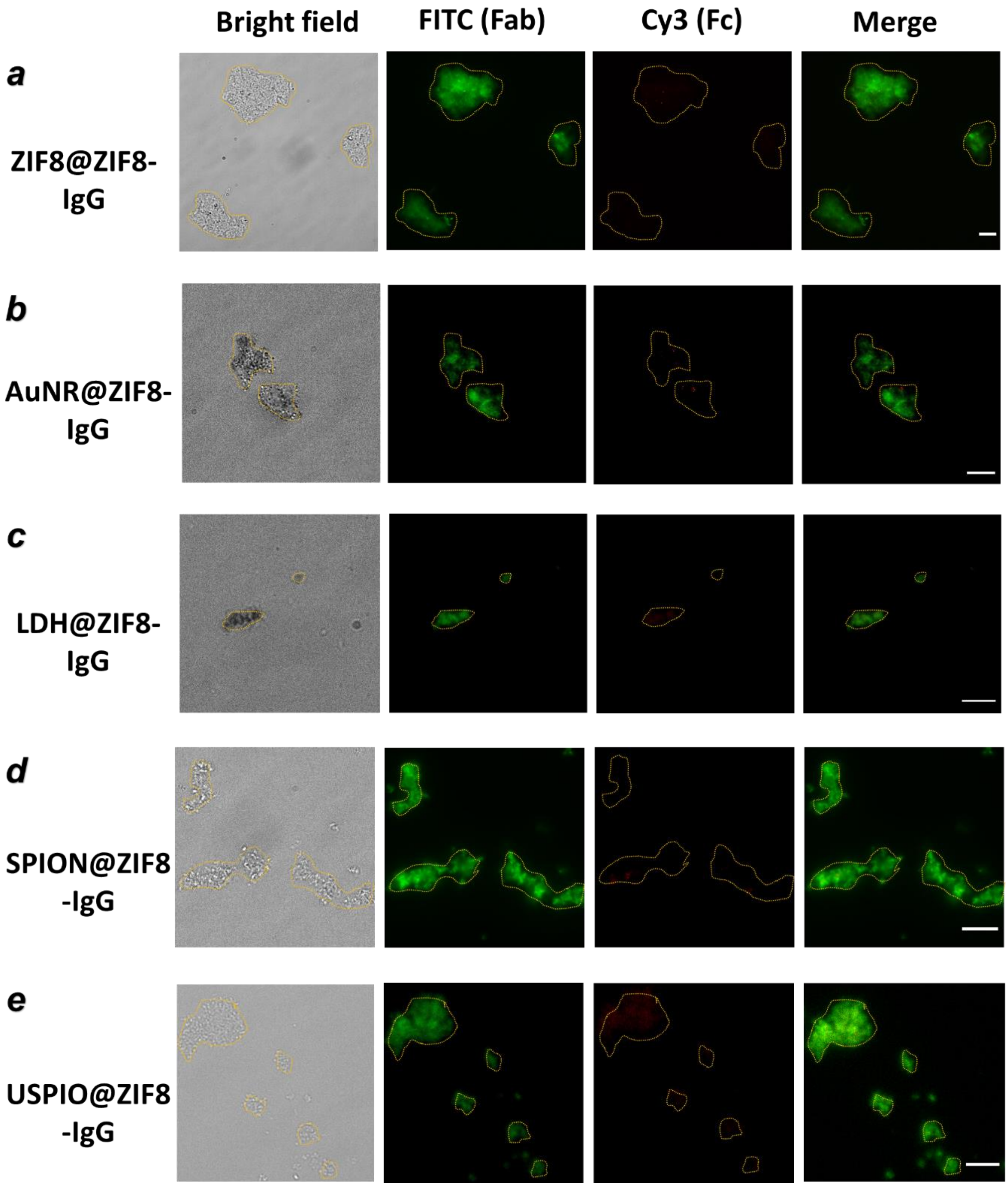

Figure S2. Fluorescence microscopy images of (a) ZIF8@ZIF8-IgG, (b) AuNR@ZIF8-IgG, (c) LDH@ZIF8-IgG, (d) USPIO@ZIF8-IgG and (e) SPION@ZIF8-IgG using secondary antibodies specifically binding to the Fab regions (FITC-labeled, green) and Fc regions (Cy3-labeled, red) (scale bar = 10 μm).

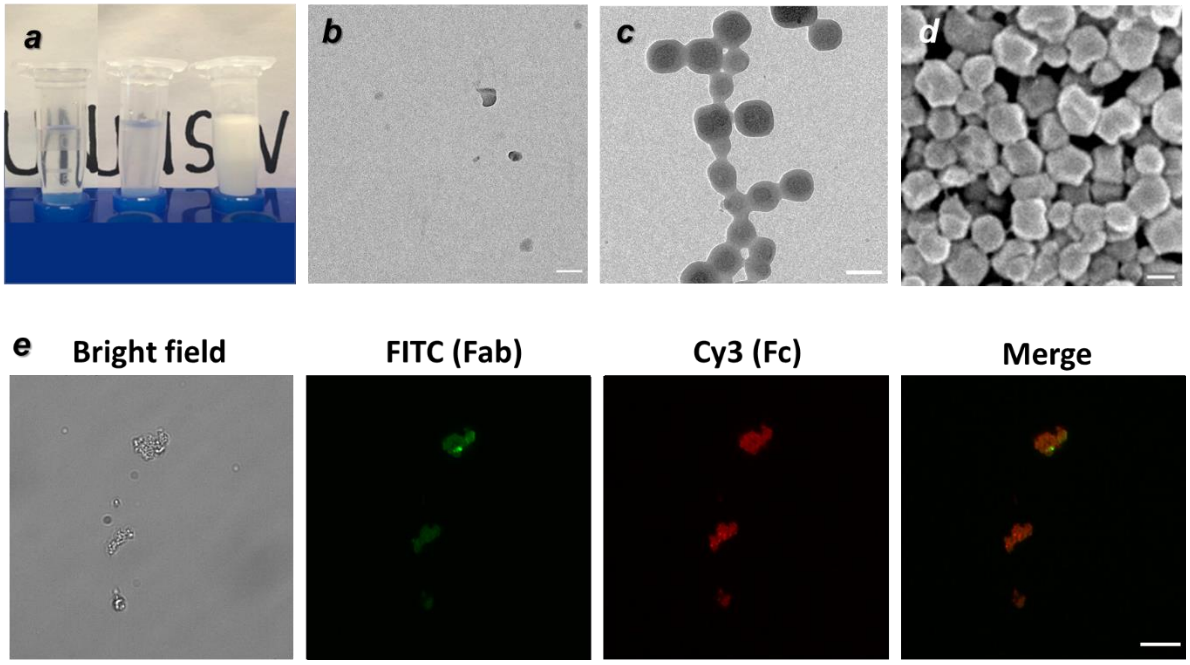

Figure S3. (a) Digital photos of IgG solution (0.25 mg mL^{-1}) (left), solution of 2-methylimidazole (20.5 mg, 0.25 M) and zinc nitrate hexahydrate (0.744 mg, 2.5 mM) (middle), and solution of 2-methylimidazole (205 mg, 2.5 M) and zinc nitrate hexahydrate (7.44 mg, 25 mM) (right). (b-c) TEM image of the sample after mixing 2-methylimidazole (20.5 mg, 0.25 M) and zinc nitrate hexahydrate (0.744 mg, 2.5 mM) (b), and the sample after mixing 2-methylimidazole (205 mg, 2.5 M) and zinc nitrate hexahydrate (7.44 mg, 25 mM), namely ZIF-8 nanoparticle (c). Scale bar = 100 nm. (d) SEM image of the sample after mixing zinc nitrate hexahydrate (0.744 mg, 2.5 mM) to a solution of nano-scaled ZIF-8 core particles containing 2-methylimidazole (20.5 mg, 0.25 M) and IgG (0.25 mg mL^{-1}), namely ZIF8@ZIF8-IgG nanoparticle. Scale bar = 100 nm. (e) Fluorescence images of the sample after dispersing ZIF-8 nanoparticles in 2-methylimidazole (205 mg, 2.5 M) containing IgG (0.25 mg mL^{-1}) followed by addition of zinc nitrate hexahydrate (7.44 mg, 25 mM). Scale bar = 10 µm.

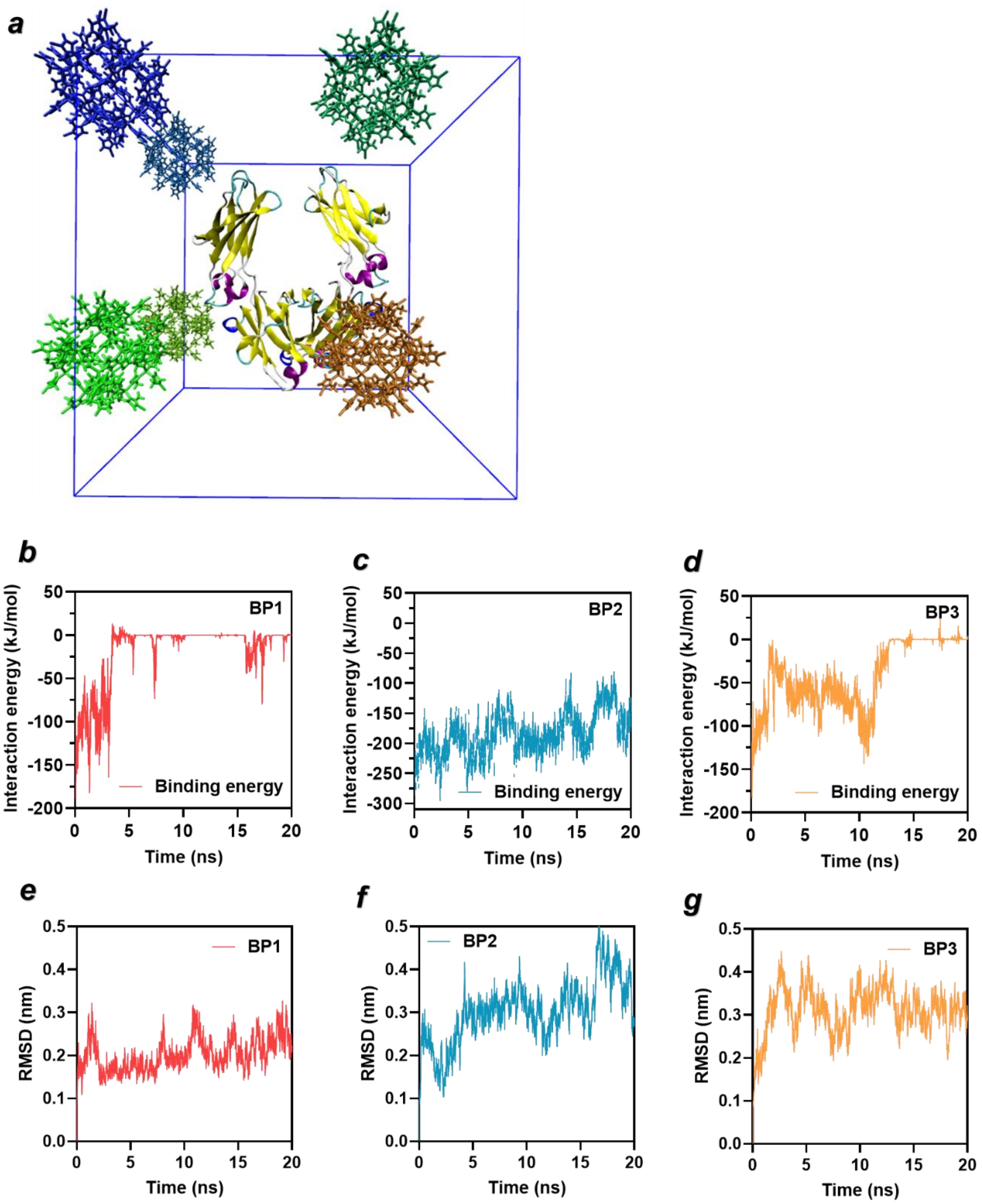

Figure S4. (a) ZIF-8 was placed at six different uncontacted positions. (b-d) Time evolution of the interaction energy between IgG and ZIF-8. (e-g) Root-mean-square deviation (RMSD) of IgG Fc.

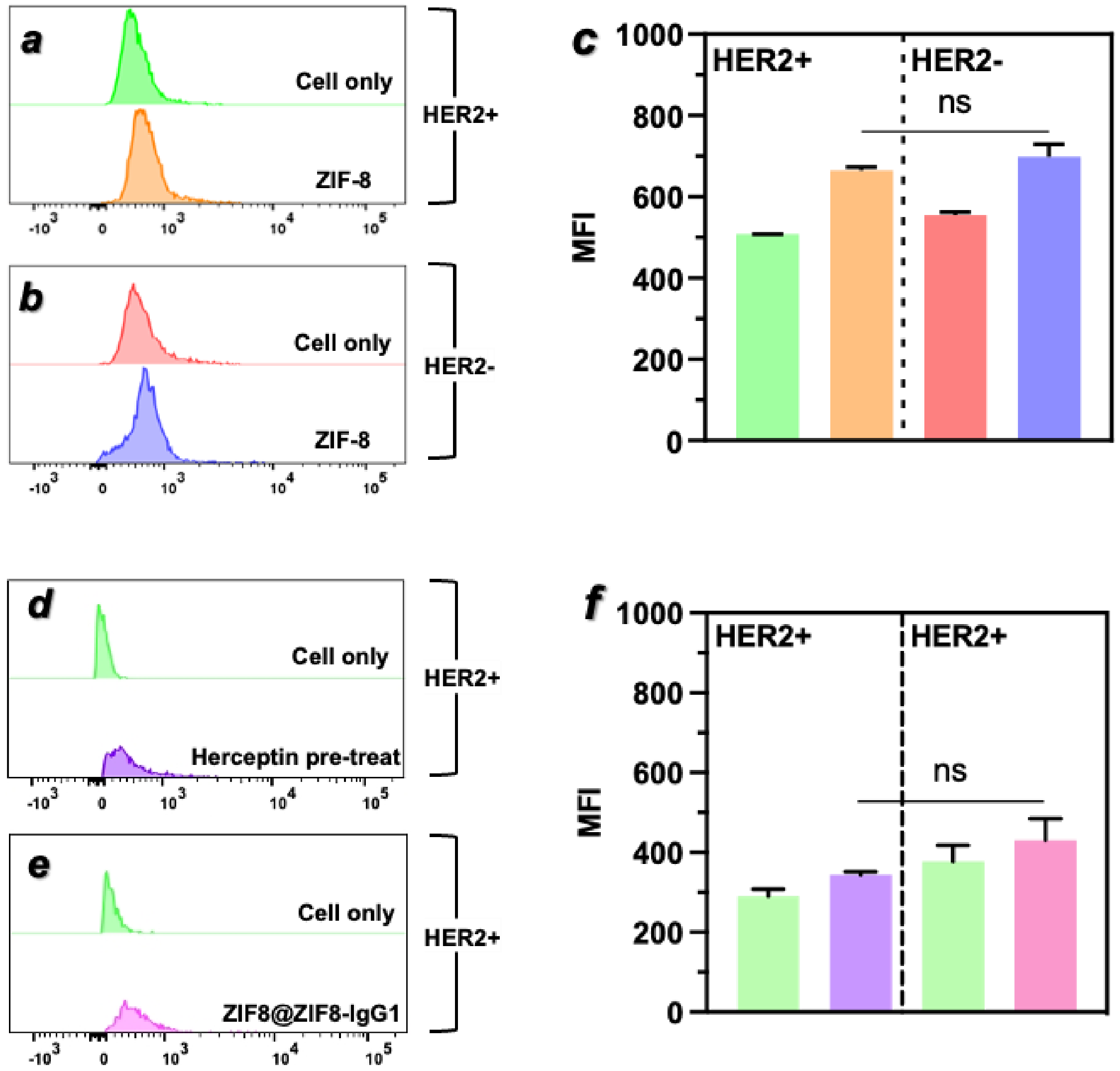

Figure S5. Cell targeting ability of ZIF8. (a, b) Flow cytometry analysis of BT-474 (HER+) and MDA-MB-231 (HER-) cells after treatment of ZIF-8 (labeled with FITC), using BT-474 and MDA-MB-231 cells without any treatment as controls and (c) their corresponding medium fluorescence intensity (MFI) analysis (mean ± SD, n = 2). (d-f) Cell targeting ability of Herceptin pre-treatment and isotype control. (d, e) Flow cytometry analysis of BT-474 (HER+) cells after pre-treatment of free Herceptin followed by ZIF8@ZIF8-HER2 (labeled with FITC) treatment, and with isotype control ZIF8@ZIF8-IgG1 treatment, and (f) their corresponding median fluorescence intensity (MFI) analysis (mean ± SD, n = 2). *ns*: no significant difference.

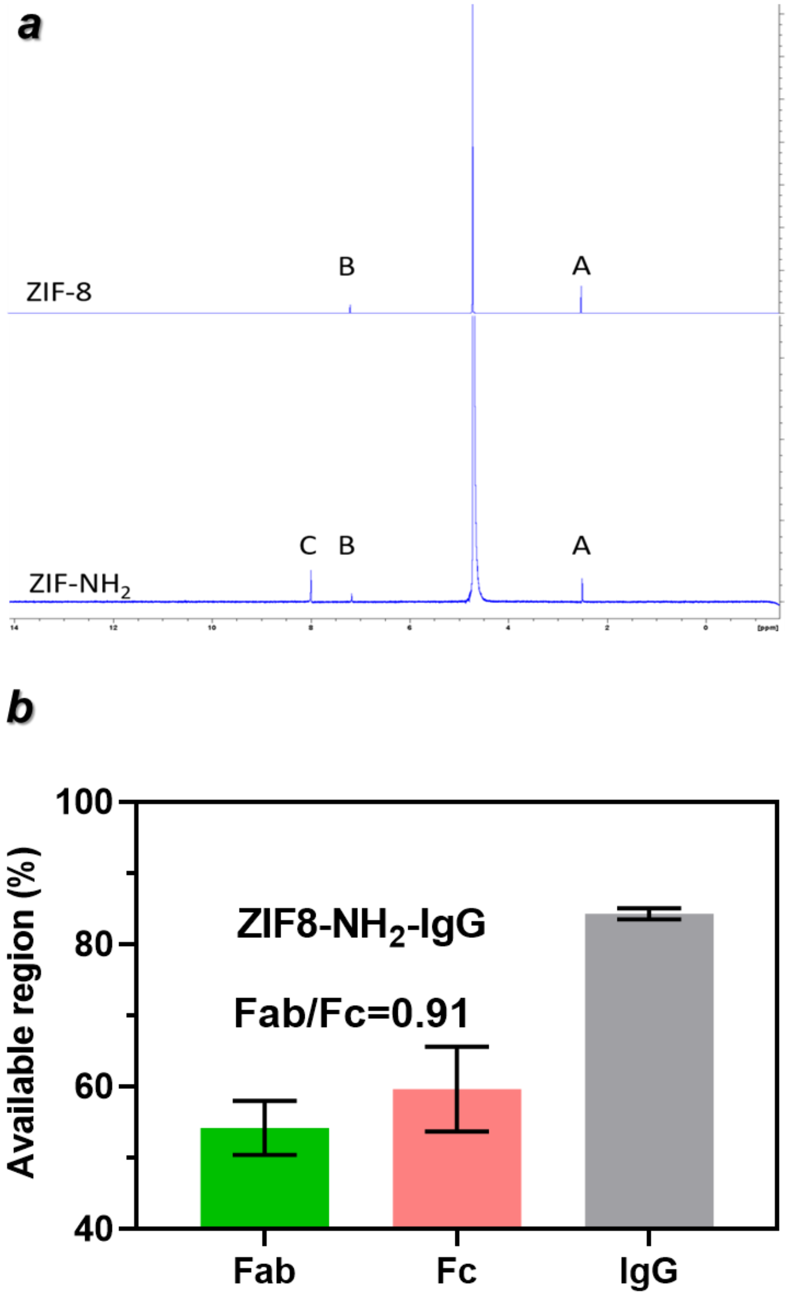

Figure S6. (a) ^{1}H NMR spectra of ZIF-8 and ZIF-NH_2. The ZIF-8 spectra exhibited peaks at 2.4 ppm (A) and 6.9 ppm (B), which correspond to methyl and methine groups of HmIm. Atz exchange resulted in a new peak at 7.8 ppm (C), which corresponds to the methine peak of Atz. NH_2 loading ratio=C/(B+C)=1/1.2321=81.2%. (b) Secondary antibody binding assay of ZIF8-NH_2-IgG. n = 2.

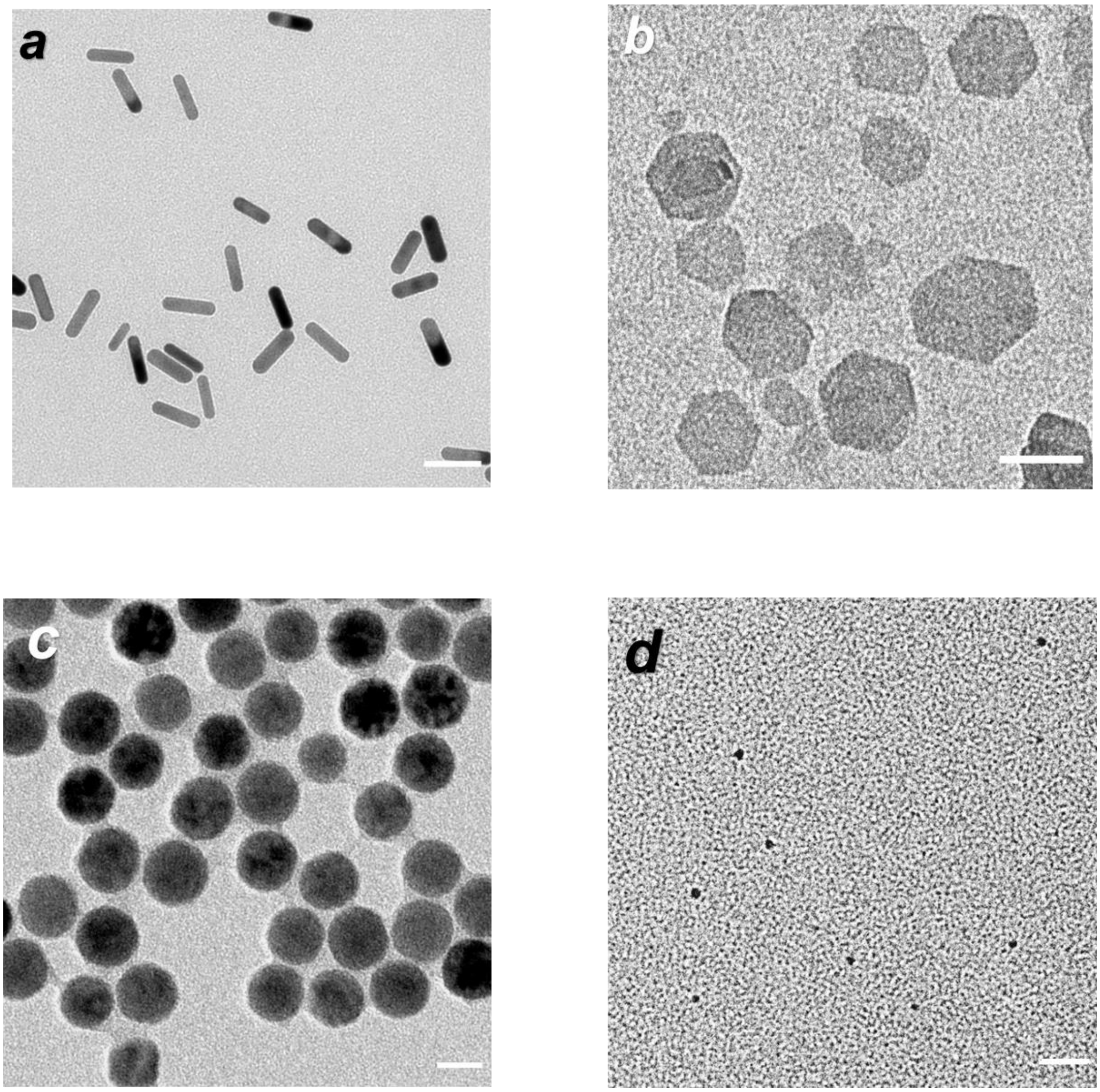

Figure S7. TEM images of (a) PSS-AuNR (scale bar = 50 nm), (b) LDH-ICG (scale bar = 50 nm), (c) SPION (scale bar = 20 nm), and (d) USPIO (scale bar = 10 nm).

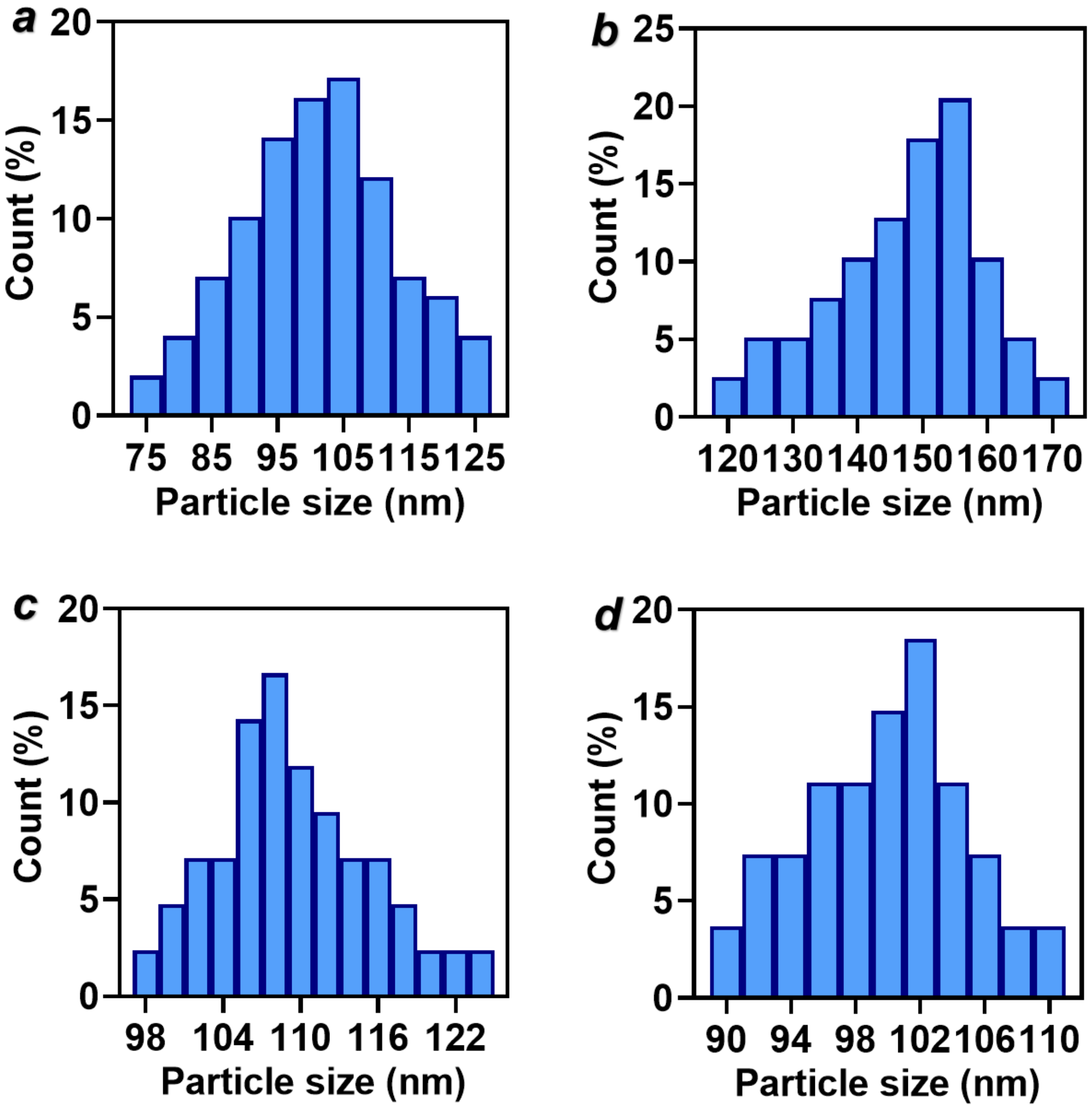

Figure S8. Particle size distribution of (a) AuNR@ZIF8-IgG, (b) LDH@ZIF8-IgG, (c) SPION@ZIF8-IgG, and (d) USPIO@ZIF8-IgG by frequency counting.

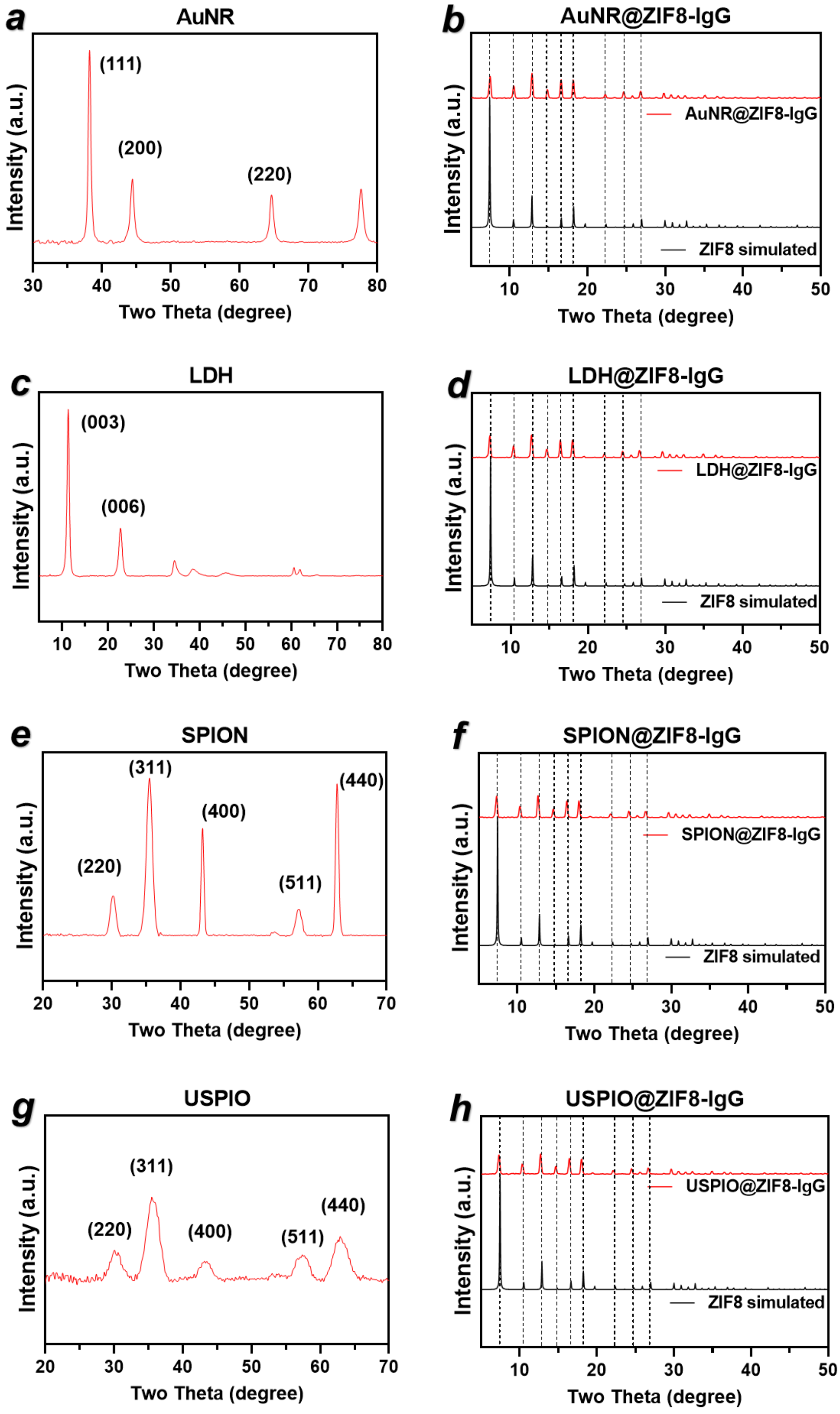

Figure S9. Powder XRD patterns of (a) AuNR, (b) AuNR@ZIF8-IgG, (c) LDH, (d) LDH@ZIF8-IgG, (e) SPION, (f) SPION@ZIF8-IgG, (g) USPIO, and (h) USPIO@ZIF8-IgG.

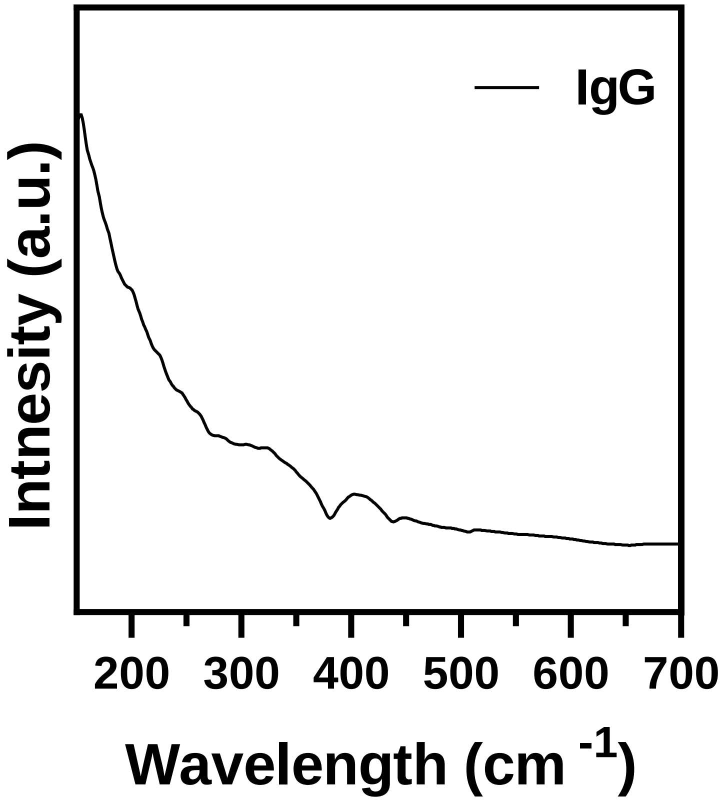

Figure S10. THz-FIR radiation of human IgG, showing no distinct feature in the tested wavenumber range.

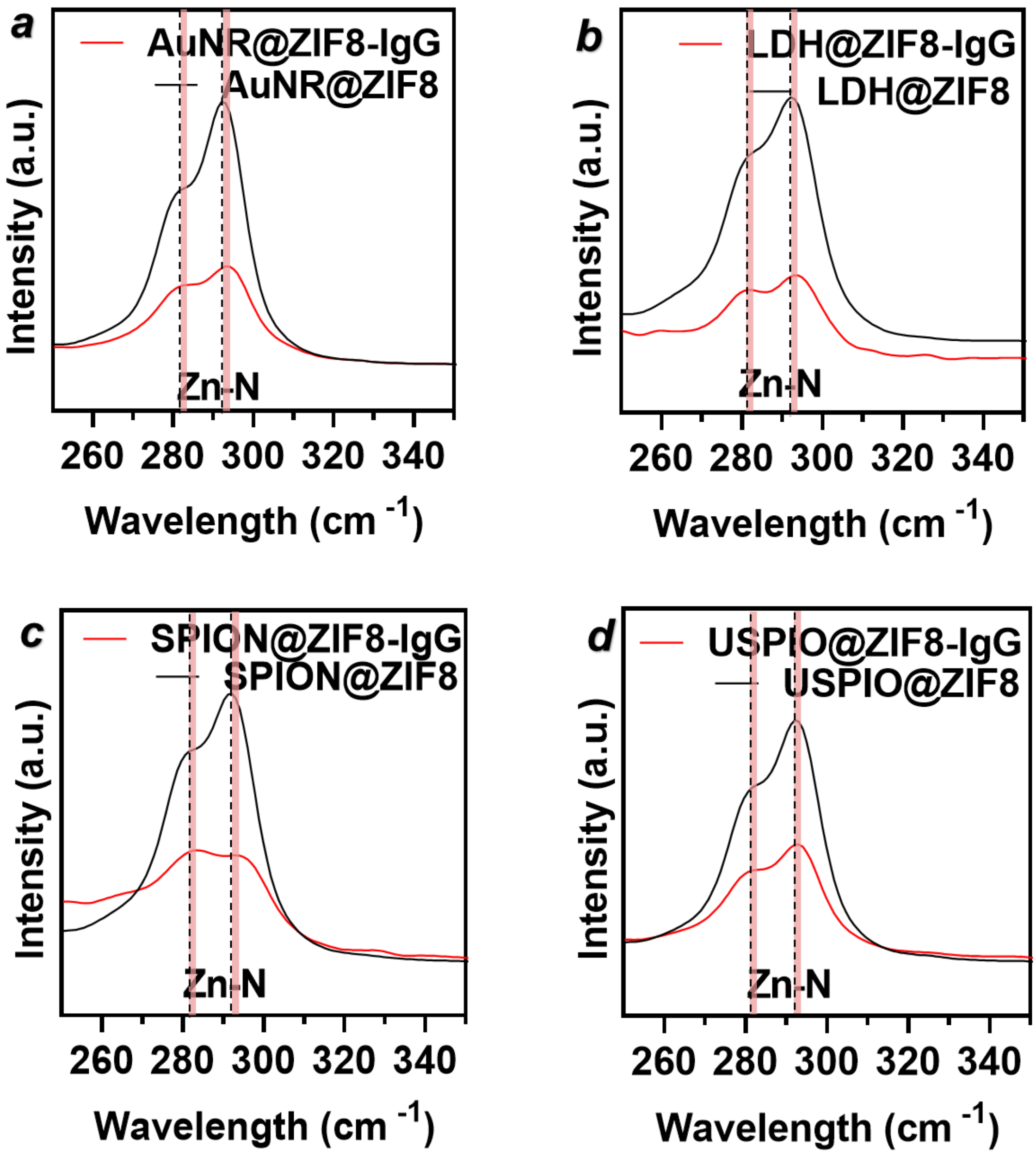

Figure S11. Magnified THz-FIR spectroscopy of (a) AuNR@ZIF8-IgG and AuNR@ZIF8, (b) LDH@ZIF8-IgG and LDH@ZIF8, (c) SPION@ZIF8-IgG and SPION@ZIF8, and (d) USPIO@ZIF8-IgG and USPIO@ZIF8

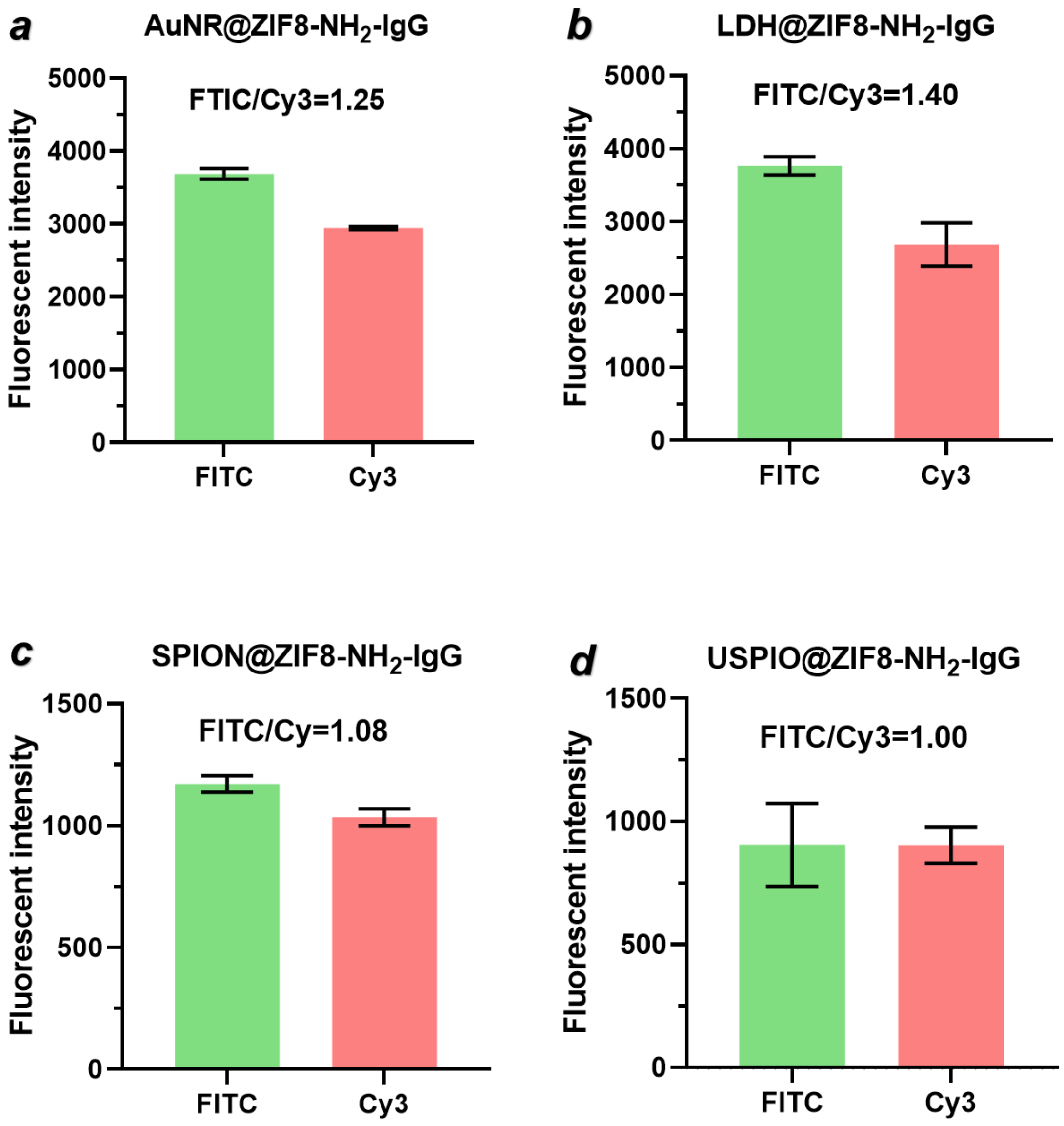

Figure S12. Fluorescence intensity of (a) AuNR@ZIF8-NH_2-IgG, (b) LDH@ZIF8-NH_2-IgG, (c) SPION@ZIF8-NH_2-IgG and (d) USPIO@ZIF8-NH_2-IgG (mean ± SD, n = 2) via flow cytometry.

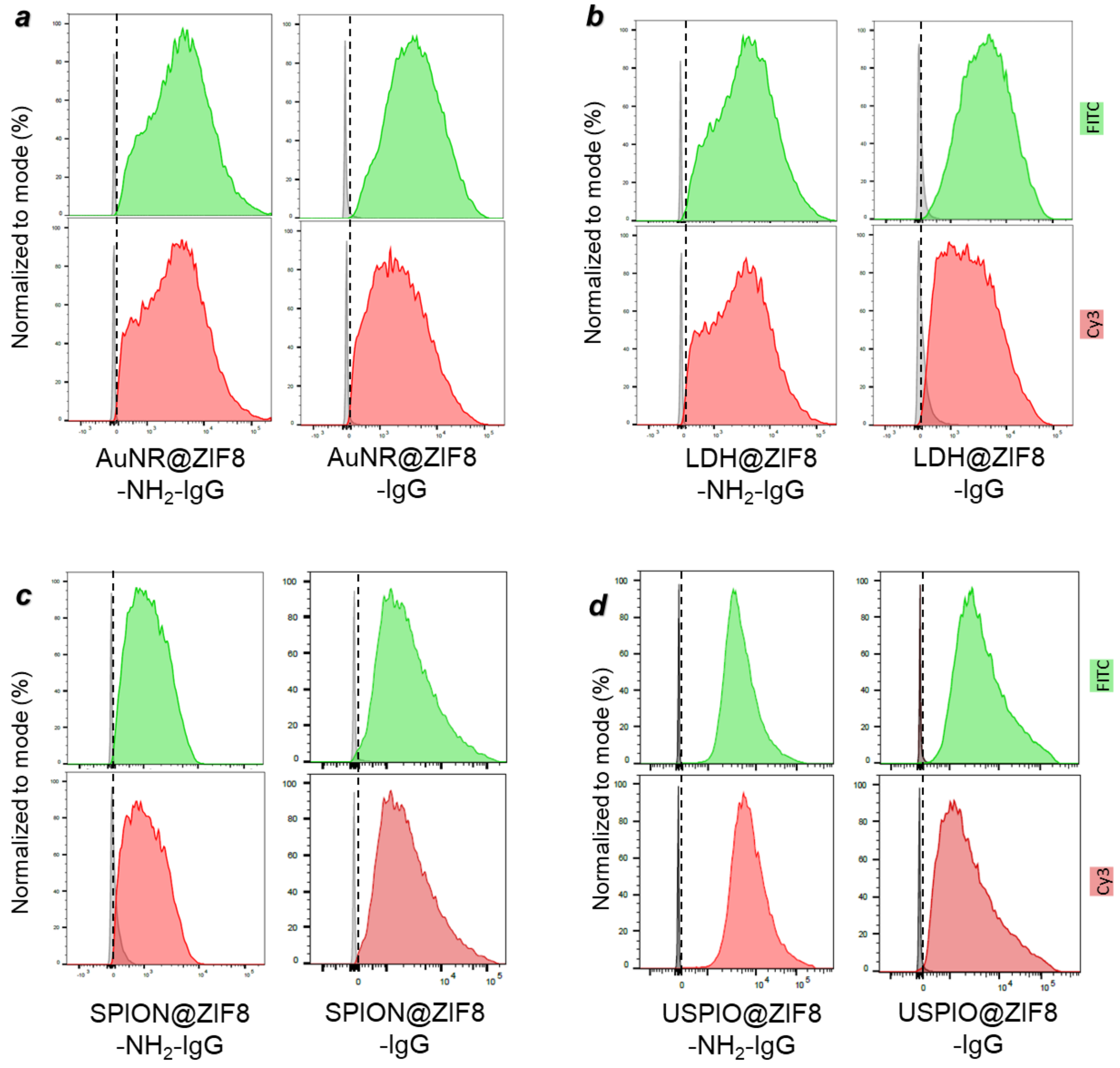

Figure S13. Representative FACS files showing the fluorescence intensity of (a) AuNR@ZIF8-NH_2-IgG and AuNR@ZIF8-IgG, (b) LDH@ZIF8-NH_2-IgG and LDH@ZIF8-IgG, (c) SPION@ZIF8-NH_2-IgG and SPION@ZIF8-IgG, and (d) USPIO@ZIF8-NH_2-IgG and USPIO@ZIF8-IgG.

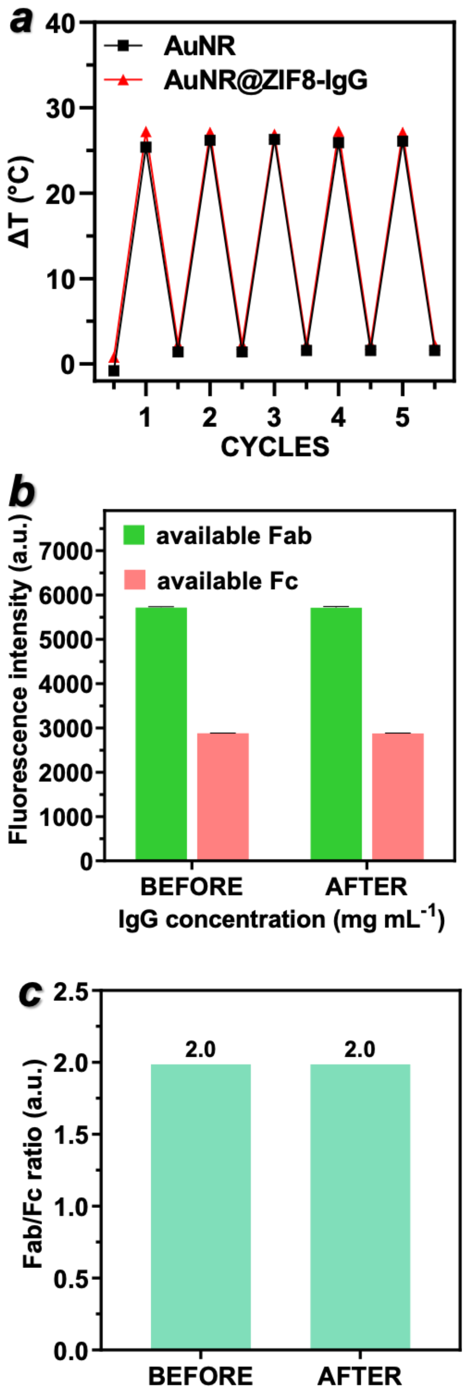

Figure S14. (a) Temperature variation of AuNR and AuNR@ZIF8-IgG ([Au] = 40 μg mL^{-1}) over five cycles, irradiated by an 808 nm laser at a power density of the 1.0 W cm^{-2}. (b) Fluorescence intensity of available Fab and available Fc regions and (c) the corresponding fluorescence intensity ratio of Fab/Fc of ZIF8@ZIF8-IgG before and after heating at 50 ˚C for 10 min at concentration of 0.25 mg mL^{-1} IgG.

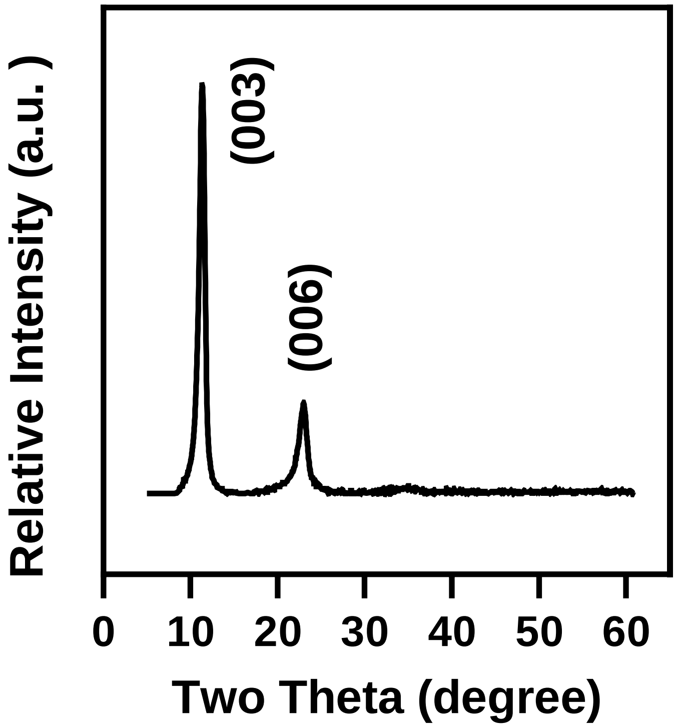

Figure S15. Film XRD pattern of LDH nanoparticles.

Samples	Thickness in c-axis (nm)	d-spacing (Å)	FWHM (°)	Layers
LDH	11.3	5.2	0.699	21

Table S1. Summary of LDH nanoparticles.

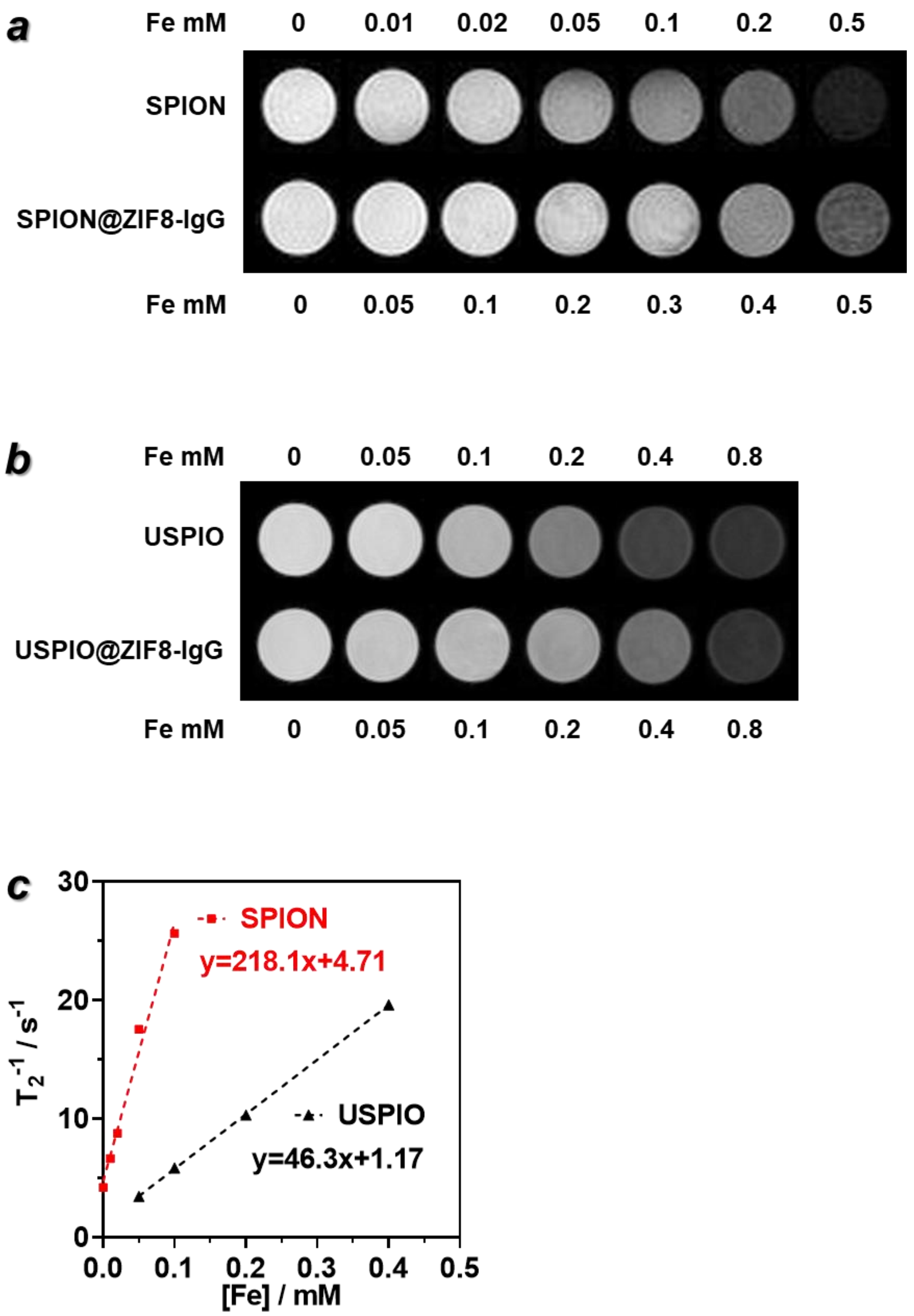

Figure S16. (a) T_2-MRI images of SPION and SPION@ZIF8-IgG. (b) T_2-MRI images of USPIO and USPIO@ZIF8-IgG. (c) Plot of T_2^{-1} versus Fe concentration of SPION and USPIO after incubation with pH 5 buffer solution at 37 °C for 24 h.
